# Unbalanced networks and disturbed kinetics of serum soluble mediators associated with distinct disease outcomes in severe COVID-19 patients

**DOI:** 10.3389/fimmu.2022.1004023

**Published:** 2022-11-14

**Authors:** Gabriela Profírio Jardim-Santos, Heidi Luise Schulte, Patricia Shu Kurizky, Ciro Martins Gomes, Otávio Tolêdo Nóbrega, Eliana Teles de Gois, Maíra Rocha Machado de Carvalho, Francielle Pulccinelli Martins, André Moraes Nicola, Cleandro Pires de Albuquerque, Laila Salmen Espindola, Luciana Ansaneli Naves, Alexandre Anderson de Sousa Munhoz Soares, Patrícia Albuquerque, Wagner Fontes, Laurence Rodrigues do Amaral, Matheus de Souza Gomes, Pedro Luiz Lima Bertarini, Joaquim Pedro Brito-de-Sousa, Ana Carolina Campi-Azevedo, Vanessa Peruhype-Magalhães, Andrea Teixeira-Carvalho, Valéria Valim, Olindo Assis Martins-Filho, Licia Maria Henrique da Mota

**Affiliations:** ^1^ Programa de Pós-Graduação em Ciências Médicas, Universidade de Brasília, Brasília, Distrito Federal, Brazil; ^2^ Serviço de Dermatologia, Hospital Universitário de Brasília, Universidade de Brasília, Brasília, Distrito Federal, Brazil; ^3^ Programa de Pós-Graduação em Patologia Molecular, Universidade de Brasília, Brasília, Distrito Federal, Brazil; ^4^ Unidade de Clínica Médica, Hospital Regional do Gama, Brasília, Distrito Federal, Brazil; ^5^ Unidade de Clínica Médica, Hospital Regional da Asa Norte, Brasília, Distrito Federal, Brazil; ^6^ Faculdade de Medicina, Universidade de Brasília, Brasília, Distrito Federal, Brazil; ^7^ Hospital Universitário de Brasília, Universidade de Brasília, Brasília, Distrito Federal, Brazil; ^8^ Programa de Pós-Graduação em Ciências da Saúde, Universidade de Brasília, Brasília, Distrito Federal, Brazil; ^9^ Faculdade UnB Ceilândia, Universidade de Brasília, Brasília, Distrito Federal, Brazil; ^10^ Laboratório de Bioquímica e Química de Proteínas, Departamento de Biologia Celular, Instituto de Ciências Biológicas, Universidade de Brasília, Brasília, Distrito Federal, Brazil; ^11^ Laboratório de Bioinformática e Análises Moleculares, Universidade Federal de Uberlândia, Patos de Minas, Minas Gerais, Brazil; ^12^ Laboratório de Tecnologias Urbanas e Rurais, Faculdade de Engenharia Elétrica, Universidade Federal de Uberlândia, Patos de Minas, Minas Gerais, Brazil; ^13^ Instituto René Rachou, Fundação Oswaldo Cruz (FIOCRUZ-Minas), Belo Horizonte, Minas Gerais, Brazil; ^14^ Hospital Universitário Cassiano Antônio Moraes, Programa de Pós-Graduação em Saúde Coletiva, Universidade Federal do Espírito Santo, Vitória, Espírito Santo, Brazil

**Keywords:** serum soluble mediators, COVID-19, cytokines, chemokines, kinetics, immune response, biomarkers

## Abstract

The present study applied distinct models of descriptive analysis to explore the integrative networks and the kinetic timeline of serum soluble mediators to select a set of systemic biomarkers applicable for the clinical management of COVID-19 patients. For this purpose, a total of 246 participants (82 COVID-19 and 164 healthy controls – HC) were enrolled in a prospective observational study. Serum soluble mediators were quantified by high-throughput microbeads array on hospital admission (D0) and at consecutive timepoints (D1-6 and D7-20). The results reinforce that the COVID-19 group exhibited a massive storm of serum soluble mediators. While increased levels of CCL3 and G-CSF were associated with the favorable prognosis of non-mechanical ventilation (nMV) or discharge, high levels of CXCL10 and IL-6 were observed in patients progressing to mechanical ventilation (MV) or death. At the time of admission, COVID-19 patients presented a complex and robust serum soluble mediator network, with a higher number of strong correlations involving IFN-γ, IL-1Ra and IL-9 observed in patients progressing to MV or death. Multivariate regression analysis demonstrates the ability of serum soluble mediators to cluster COVID-19 from HC. Ascendant fold change signatures and the kinetic timeline analysis further confirmed that the pairs “CCL3 and G-CSF” and “CXCL10 and IL-6” were associated with favorable or poor prognosis, respectively. A selected set of systemic mediators (IL-6, IFN-γ, IL-1Ra, IL-13, PDGF and IL-7) were identified as putative laboratory markers, applicable as complementary records for the clinical management of patients with severe COVID-19.

## Introduction

The Severe Acute Respiratory Syndrome Coronavirus 2 (SARS-CoV-2) disease (COVID-19) has been identified as an infectious disease affecting the human respiratory system. According to the World Health Organization, over 539 million COVID-19 cases, including approximately 6.3 million deaths, have been confirmed globally since the SARS-CoV-2 outbreak in 2019 (1). The clinical presentation of COVID-19 resembles a respiratory infection with morbidity ranging from a mild cold-like manifestation to severe viral pneumonia that can lead to a potentially fatal acute respiratory distress syndrome (2).

The exact pathophysiology of COVID-19 remains under constant investigation; however, the disease pathogenesis has been strongly associated with an unbalanced immune response (3–5). A dysregulated systemic inflammatory response, known as cytokine storm or cytokine release syndrome, was observed in severe COVID-19 patients and correlated with a poor outcome. The massive release of pro-inflammatory cytokines by immune and non-immune effector cells contributes to SARS-CoV-2 pulmonary inflammation, extensive lung damage, and hyper-coagulation, adding to the severity of the disease. A number of studies demonstrated that patients with poor outcomes often present elevated levels of several pro-inflammatory mediators, including chemokines (e.g. CXCL10, CCL2, CCL5, and CCL3) and cytokines (e.g. IL-6, TNF-α, IL-1, INF-γ, and IL-1β) (3–5).

The production and release of the pro-inflammatory mediators, regulatory molecules, and cell growth factors are strictly structured and temporally coordinated to guarantee immune system homeostasis. Dysregulation of the immune mediator microenvironment usually results in unbalanced immune responses that may lead to hyper-activation, an increase in the pro-inflammatory profile that further contributes to the worsening of COVID-19 clinical manifestations. In some cases, immune dysregulation may lead to the requirement of intensive care and mechanical ventilation or disease progression to death. This dysfunction can not only refer to excessive or insufficient levels but also to the production and release of immune mediators in an inappropriate timeline. As such, a broad understanding of the kinetic timeline of distinct immune mediators in patients with severe COVID-19 is still relevant to explore the complex multifactorial network underlying the disease course in order to provide novel insight into prognostic and therapeutic implications (3, 5–8). Additionally, the search for potential laboratory markers can provide supplementary objective information that may substantially improve patient care (9).

In this context, exploratory investigations employing distinct models of descriptive analysis to characterize the panoramic profile of serum soluble mediators in patients with severe COVID-19 are relevant to identify novel biomarkers to understand the mechanisms underlying distinct disease outcomes. Our findings reinforce that as well as the well-known pro-inflammatory storm, an unbalanced integrative network, increased orders of magnitude and disturbed kinetics of serum soluble mediators were associated with distinct disease outcomes in patients with severe COVID-19. Moreover, a selected set of systemic mediators were identified as putative laboratory markers, applicable as complementary records for the clinical management of severe COVID-19 patients.

The aim of the present study was to carry out a prospective observational investigation to quantify, by high-throughput multiplex bead array, the serum levels of serum soluble mediators in patients with severe COVID-19, at hospital admission (D0) and in consecutive timepoints, including the first (D1-6) and the second week (D7-20) of hospitalization. The main goal was to verify the association between the profile of serum levels of serum soluble mediators and the disease outcome towards Non-Mechanical Ventilation (nMV) vs Mechanical Ventilation and Discharge vs Death.

## Material and methods

### Study population

This is a prospective observational study conducted between May 2020 and September 2020 during peak circulation of the SARS-CoV-2 strains B.1.1.28 and B.1.1.33, in the COVID-19 pandemic in the Federal District of Brazil. A total of 246 participants were considered as a non-probabilistic convenience sampling, including patients with severe acute SARS-CoV-2 infection (COVID-19, n=82) together with age- and sex-matched non-infected pre-pandemic healthy controls (HC=164).

Critically ill COVID-19 patients were enrolled upon admission at three public hospitals participating in a large research protocol named the TARGET project (10). The COVID-19 group comprised 48 males and 34 females, aged 23 to 76 years, with a median age of 50 years. The COVID-19 patients were further categorized into subgroups according to disease outcome, referred to as: Non-Mechanical Ventilation (nMV, n=71) vs Mechanical Ventilation (MV, n=11) and Discharge (n=75) vs Death (n=7). The inclusion criteria consisted of severe COVID-19 diagnosis (with positive RT-PCR for SARS-CoV-2) and the ability to provide study participation consent. Critically ill COVID-19 cases comprised patients with respiratory distress, respiratory rate ≥30/min or oxygen saturation ≤93% on room air. For all patients who met the inclusion criteria, demographic and clinical data were obtained from medical records at enrollment. Dyspnea, cough, and fever were the most frequent symptoms. The most frequent comorbidity was hypertension and diabetes. Exclusion criteria constituted: organic, functional, or oligophrenic mental illness that affected the quality of the information provided by the patient. All COVID-19 patients received corticosteroid (methylprednisolone or dexamethasone) and antibiotic (ceftriaxone, azithromycin or ampicillin/sulbactam) therapy together with anticoagulant medicine (low molecular weight heparin). Patients were recruited on hospital admission (D0) and monitored for 20 consecutive days.

A total of 164 age and sex-matched healthy subjects were included as the reference control group (HC). The HC group comprised 96 males and 68 females, aged from 23 to 76 years (median = 50 years). This control group, composed of non-infected pre-pandemic healthy controls (HC), was selected as a non-probabilistic convenience sampling from a biorepository maintained at *Grupo Integrado de Pesquisas em Biomarcadores, Instituto René Rachou*, Fundação Oswaldo Cruz (FIOCRUZ-Minas), Belo Horizonte, Brazil.

All participants enrolled in the present investigation signed an informed consent form in accordance with the Declaration of Helsinki and Resolution 466/2012 of the Brazilian National Health Council for research involving human subjects. This study was recorded in the Brazilian Registry of Clinical Trials Platform (ReBEC, RBR-62zdkk) and approved by the National Commission for Ethics in Research in Brazil (CONEP, CAAE 30846920.7.0000.0008).

### Biological samples

Whole blood samples (10 mL) were collected from each participant in vacuum tubes without anticoagulant by venipuncture at three consecutive timepoints: on hospital admission (D0), during the first week (D1-6), and from the second week (D7-20) of hospitalization. Serum samples were obtained by centrifugation (1400 x g, 10 min, 4°C) of original samples within 12 h after blood collection. The serum specimens were aliquoted and stored at -80 °C until used for the analysis of serum soluble mediators.

### Analysis of serum soluble mediators

Serum soluble mediators were measured by a high-throughput microbeads multiplex array (Bio-Plex Pro™ Human Cytokine 27-plex Assay, Bio-Rad Laboratories, Hercules, CA, USA). The concentrations of chemokines (CXCL8; CCL11; CCL3; CCL4; CCL2; CCL5; CXCL10), pro-inflammatory cytokines (IL-1β; IL-6; TNF-α; IL-12; IFN-γ; IL-15; IL-17), regulatory cytokines (IL-1Ra; IL-4; IL-5; IL-9; IL-10; IL-13) and growth factors (FGF-basic; PDGF; VEGF; G-CSF; GM-CSF; IL-2; IL-7) were quantified according to the manufacturer’s instructions. A trained technician performed the microbeads multiplex assays in parallel batches at the flow cytometry facility at FIOCRUZ-Minas. Quantitative analysis of serum soluble mediators was performed using a 5-parameter logistic curve fit from standard curves, with results expressed as pg/mL.

### Statistical analysis

Several software were used to carry out the data analysis with the purpose of getting the most from each statistical packages. Although some software have operational packages with similar functions, they also present specific functions that were relevant to accomplish a broader exploratory analysis.

Descriptive statistical analyses were performed, and the data normality test was assessed by the Shapiro-Wilk test using the Prism 8.0.2 software (GraphPad Software, San Diego, USA). Baseline levels of soluble mediators in the serum of the COVID-19 group at timepoint D0 were compared to non-infected pre-pandemic Healthy Controls (HC) using the Mann-Whitney test, considering the nonparametric distribution of all data sets. In all cases, a threshold of p<0.05 was considered for statistical significance.

Correlation analysis (Pearson and Spearman correlation tests) was used to construct correlation matrices and assemble integrative networks. Only significant correlations at p< 0.05 were considered. Correlation matrices were built using the R software (Project for Statistical Computing version 3.0.1) and the “corrplot” package. A color key scaled from −1 to +1 was used to identify positive (blue) and negative (red) correlations, with white squares indicating non-significant correlations. The Cytoscape software, an open-source platform (available at https://cytoscape.org), was used to create cluster layouts. Serum soluble mediator networks were designed using a cluster network layout to represent each serum soluble mediator category (chemokines, pro-inflammatory cytokines, regulatory cytokines, and growth factors).

The MATLAB software was used for Principal Component Analysis (PCA) and regression analysis.

The PCA data was assembled to verify the ability of serum soluble mediators to cluster COVID-19 from HC, as well as subgroups of COVID-19, according to disease outcome. The PCA analysis allows to reduce the data dimensionality.

The multivariate regression model was trained using MATLAB’s Regression Learner app, assigning values of 0 to COVID-19, nMV and Discharge classes and values of 1 to HC, MV and Death classes. 24 different regression models were tested with a 10-fold cross-validation method aiming to predict the 0 or 1 values from serum soluble mediator values. In each scenario, the best model was selected based on the highest R-squared (COVID vs HC - Model Boosted Tree Coarse; nMV vs MV - Model Gaussian SVM; Discharge vs Death - Model Medium Gaussian SVM).

The magnitude of change in the serum levels of soluble mediators in the COVID-19 group was calculated as the proportion ratio between the serum levels observed for each COVID-19 patient at the time of admission (D0), D1-6 and D7-20 divided by the median values reported for non-infected pre-pandemic Healthy Controls (HC). The magnitude of changes in the serum levels of soluble mediators were determined considering: decreased (≤ 3x), unaltered (0.4x - 2x) and increased (≥ 3x) levels in relation to the median values observed in the HC. Orbital graphs were generated using Microsoft Excel version 2012.

Heatmap constructs were assembled using conditional formatting in Microsoft Excel. The analysis was performed using the median values of the magnitude of change in serum soluble mediators in the COVID-19 group relative to the HC group. A color key was used to underscore the attributes with decreased (fold change <1x, towards green), unaltered (fold change =1x, black) or increased levels (fold change >1x, towards red). Timeline diagrams were employed to identify common and selective soluble mediators with decreased (≤0.3x, green) or increased (≥3x, red) levels along the kinetic timeline.

Receiver Operating Characteristic (ROC) curve analysis was performed using the MedCalc software, version 7.3.0.0 (Ostend, Belgium, URL https://www.medcalc.org/) for each soluble mediator to assess the performance indices (area under the ROC curve = AUC, Sensitivity = Se, Specificity = Sp and Likelihood ratio = LR). The AUC values were considered to pre-select soluble mediators for further analysis. A scatter plot distribution of individual values was constructed to provide the detailed performance of pre-selected serum soluble mediators.

## Results

### Overall profile of serum soluble mediators in severe COVID-19 patients at the time of hospital admission

**Figure 1:**
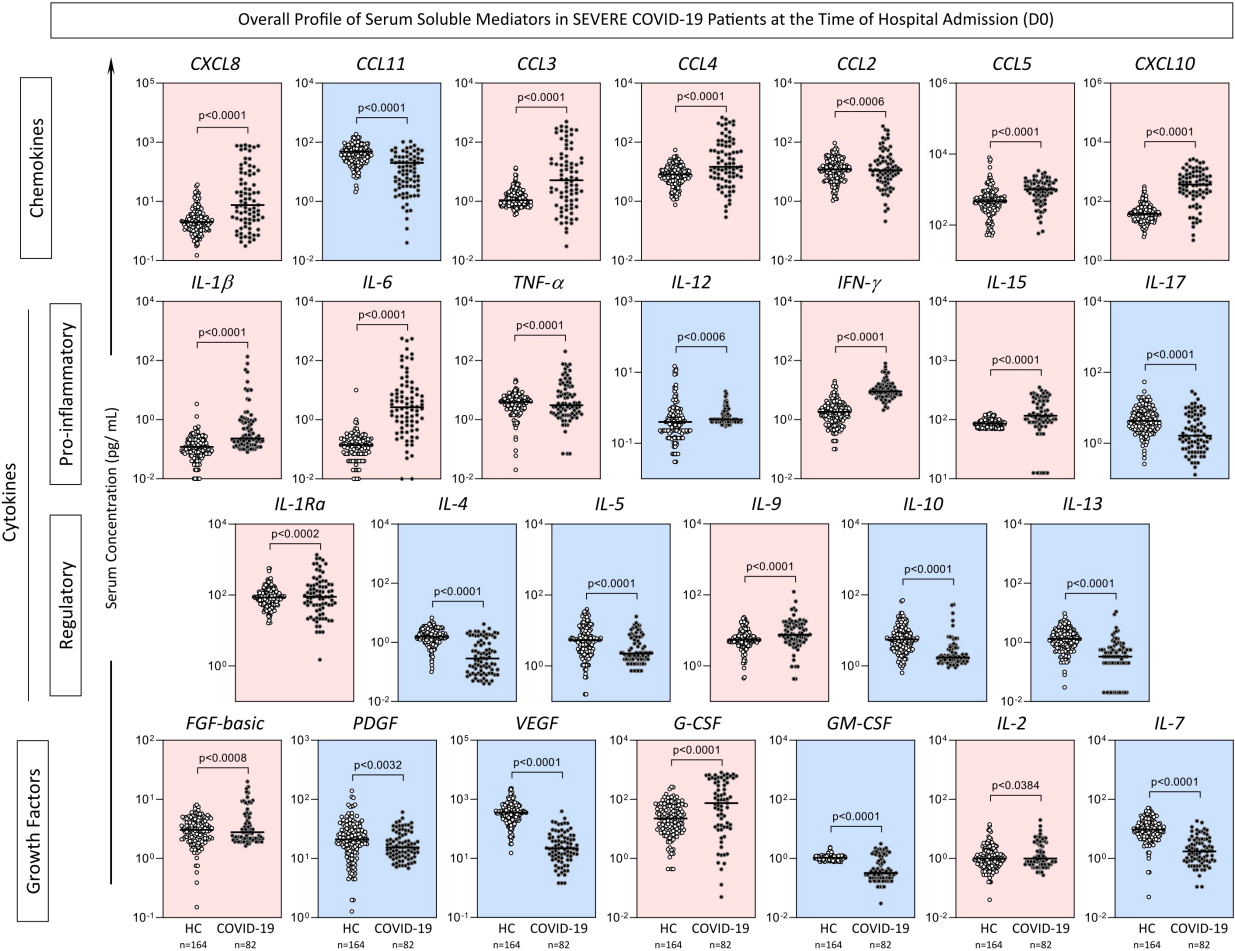
*Overall profile of serum soluble mediators in severe COVID-19 patients on hospital admission (D0).* Serum levels of chemokines (CXCL8, CCL11, CCL3, CCL4, CCL2, CCL5, CXCL10), pro-inflammatory cytokines (IL-1β, IL-6, TNF-α, IL-12, IFN-γ, IL-15, IL -17), regulatory cytokines (IL-1Ra, IL-4, IL-5, IL-9, IL-10, IL-13) and growth factors (FGF-basic, PDGF, VEGF, G-CSF, GM- CSF, IL-2, IL-7) were measured in COVID-19 patients on hospital admission (D0, n=82) and non-infected pre-pandemic Healthy Controls (HC, n=164) by high-throughput multiplex bead array as described in Material and Methods. The results are presented as scattering distribution of individual values (pg/mL) in log scale with lines showing the median values. Comparative analysis between COVID-19 vs HC was performed by Mann-Whitney test and significant differences at p<0.05 underscored by connecting lines and p values provided in the figure. Colored backgrounds highlight decreased (blue) or increased (pink) levels of serum soluble mediators in COVID-19 as compared to HC.

The levels of serum soluble mediators (chemokines, pro-inflammatory or regulatory cytokines, and growth factors) in severe COVID-19 patients were quantified by a high-throughput multiplex bead array at the time of hospital admission (D0) and compared to the levels observed in non-infected pre-pandemic healthy controls (**Figure 1**). Data analysis demonstrated that overall, the COVID-19 group exhibited a massive storm of soluble mediators in comparison to the healthy controls, characterized by increased levels of CXCL8, CCL3, CCL4, CCL2, CCL5, CXCL10, IL-1β, IL-6, TNF-α, IFN-γ, IL-15, IL-1Ra, IL-9, FGF-basic, G-CSF and IL-2; and decreased levels of CCL11, IL-12, IL-4, IL-5, IL-10, IL-13, PDGF, VEGF, GM-CSF and IL-7.

Additional analyses compared the levels of serum soluble mediators in COVID-19 patients classified according to disease outcome, referred to as: Non-Mechanical Ventilation (nMV) vs Mechanical Ventilation (MV) and Discharge vs Death. The comparative analysis demonstrated that while nMV and Discharge presented higher levels of CCL3 and G-CSF, MV and Death exhibited higher CXCL10 and IL-6 levels, in addition to lower IL-10 levels (**Table 1**).

**Table 1:** Panoramic profile of serum soluble mediators measured on hospital admission (D0) in SEVERE COVID-19 patients according to disease outcome.

Parameters	COVID-19 Subgroups			
	nMV (n=71)	MV (n=11)	Discharge (n=75)	Death (n=7)	
CXCL8	8.9 (1.7-91.2)	8.9 (4.3-294)	10.2 (2.1-88.4)	7.3 (3.6-289)	
CCL11	11.1 (3.2-32.2)	5.5 (0.6-21.8)	10.4 (3.3-31.7)	4.5 (1.1-16.2)	
**CCL3**	↑ 5.9 (0.8-31.3)*	2.2 (1.1-8.5)	↑ 5.7 (1.1-29)*	2.5 (1.3-147)	
CCL4	19.1 (6.1-85.0)	11.1 (5.7-72.7)	18.9 (6.4-81.3)	12.2 (6.2-198)	
CCL2	10.4 (4.9-24.2)	11.8 (4.7-54.4)	11.3 (5.6-23.0)	10.9 (4.6-78.9)	
CCL5	1.010 (477-1,569)	854 (514-1,650)	962 (480-1,558)	863 (683-1,560)	
**CXCL10**	339 (146-711)	↑ 557 (249-1.425)*	353 (151-716)	↑ 555 (296-1,904)*	
IL-1β	0.3 (0.1-0.9)	0.2 (0.1-0.5)	0.3 (0.1-0.9)	0.2 (0.2-1.0)	
**IL-6**	2.0 (0.5-7.2)	↑ **5.4 (2.5-10.9)** *	2.1 (0.5-7.1)	↑ 4.2 (1.8-65.9)*	
TNF-α	3.4 (1.4-13.1)	2.7 (1.5-8.8)	3.5 (1.5-11.9)	1.6 (1.6-13.8)	
IL-12	0.5 (0.4-0.8)	0.5 (0.5-0.9)	0.5 (0.4-0.8)	0.5 (0.5-1.2)	
IFN-γ	8.9 (6.6-13.6)	9.8 (8.3-19.0)	8.9 (7.0-14.5)	9.8 (7.2-19.7)	
IL-15	117 (90.4-208)	90.4 (81.9-151)	117 (90.4-208)	106 (79.9-212)	
IL-17	1.6 (0.7-6.3)	1.3 (0.8-3.0)	1.6 (0.7-6.0)	1.3 (1.0-5.6)	
IL-1Ra	93.5 (41.3-193)	69.1 (34.3-181)	93.5 (45.2-190)	60.1 (36.1-163)	
IL-4	0.3 (0.1-1.1)	0.2 (0.1-0.3)	0.3 (0.1-1.1)	0.2 (0.1-1.1)	
IL-5	2.4 (1.6-6.6)	2.0 (1.6-4.3)	2.4 (1.6-6.4)	2.2 (1.8-6.4)	
IL-9	7.3 (4.7-15.4)	10.3 (5.4-15.0)	7.4 (5.1-13.8)	13.4 (5.4-15.1)	
**IL-10**	1.8 (1.4-2.8)	↓ 1.5 (1.2-2.1)*	1.8 (1.5-2.8)	↓ **1.3 (1.2-10.3)**	
IL-13	0.5 (0.2-0.6)	0.3 (0.2-1.3)	0.3 (0.2-0.6)	0.3 (0.1-1.6)	
FGF-basic	2.8 (2.2-5.7)	2.3 (2.1-2.9)	2.8 (2.2-5.2)	2.3 (2.1-6.1)	
PDGF	21.6 (10.3-58.8)	23.7 (8.9-38.3)	21.6 (10.0-51.8)	23.7 (13.1-37.6)	
VEGF	15.6 (11.1-26.4)	15.2 (10.7-21.3)	15.6 (11.4-25.2)	12.7 (10.7-26.5)	
**G-CSF**	↑ 97.0 (11.7-438)*	11.6 (3.6-46.7)	94.0 (13.0-416)*	11.3 (3.4-38.3)	
GM-CSF	0.4 (0.2-1.3)	0.3 (0.2-0.8)	0.4 (0.2-1.2)	0.3 (0.2-1.0)	
IL-2	1.1 (0.6-3.5)	0.8 (0.6-1.7)	1.1 (0.6-3.3)	0.8 (0.6-3.1)	
IL-7	1.8 (0.8-4.2)	1.0 (0.8-8.0)	1.8 (0.8-4.0)	1.0 (0.8-5.1)	

nMV, Non-mechanical Ventilation; MV, Mechanical Ventilation. Data are shown as median values 25^th^-75^th^ interquartile range (IQR) expressed in pg/mL. Significant differences at p<0.05 are underscored by * and highlighted by bold underline format.

### Integrative serum mediator networks in severe COVID-19 patients on hospital admission

**Figure 2:**
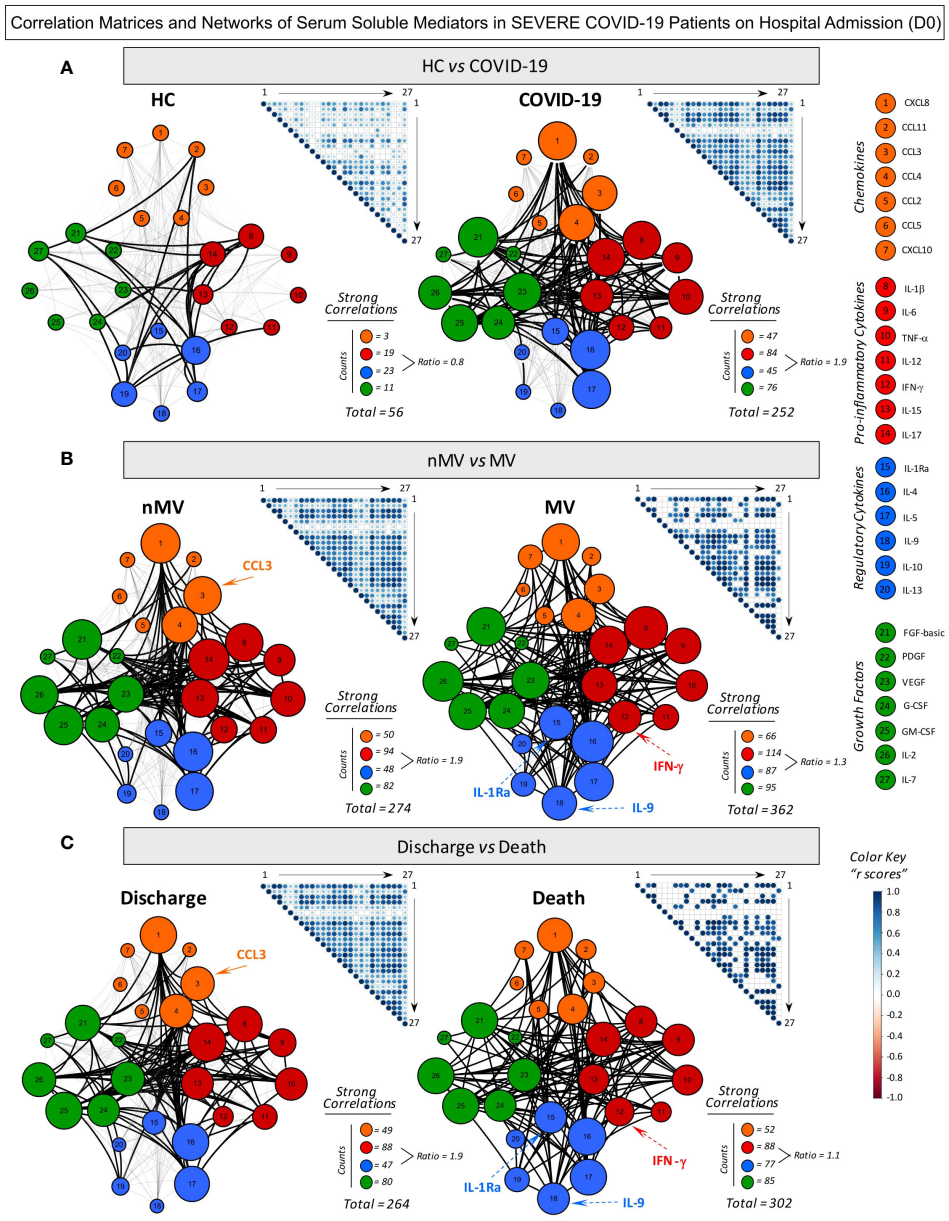
Correlation matrices and networks of serum soluble mediators in severe COVID-19 patients on hospital admission (D0). **(A)** Comprehensive correlation matrices and networks were assembled for serum chemokines, pro-inflammatory cytokines, regulatory cytokines, and growth factors observed in patients with severe COVID-19 (n=82) and non-infected pre-pandemic Healthy Controls (HC, n=164). COVID-19 patients were further categorized into subgroups according to disease outcome, referred to as: **(B)** Non-Mechanical Ventilation (nMV, n=71) vs Mechanical Ventilation (MV, n=11) and **(C)** Discharge (n=75) vs Death (n=7). The soluble mediators were measured by Luminex Bio-Plex platform as described in Material and Methods. Data analyses were performed by Pearson and Spearman rank tests and significant correlations at p<0.05 employed to build template matrices overview with each square intersection representing the “r” correlation scores between pairs of attributes. Color key scaled from −1 to +1 was used to identify positive (blue) and negative (red) correlations and white squares indicating non-significant correlations. Networks were built considering all significant correlations, using a cluster layout for each category of serum soluble mediators, with nodes representing: chemokines (Orange nodes – 1=CXCL8; 2=CCL11; 3=CCL3; 4=CCL4; 5=CCL2, 6=CCL5 and 7=CXCL10), pro-inflammatory (Red nodes – 8= IL-1β; 9=IL-6; 10= TNF-α; 11=IL-12; 12= IFN-γ; 13=IL-15 and 14=IL-17), regulatory cytokines (Blue nodes – 15=IL-1Ra; 16=IL-4; 17=IL-5; 18=IL-9; 19=IL-10 and 20=IL-13) and growth factors (Green nodes – 21=FGF-basic; 22=PDGF; 23=VEGF; 24=G-CSF; 25=GM-CSF; 26=IL-2 and 27=IL-7). Connecting edges illustrates weak/moderate (“r” scores between |0.1 to 0.67|, thin gray lines) and strong correlations (“r” scores ≥ |0.67|, thick black lines). Negative correlations are represented by dashed lines. Node sizes are proportional to the number of strong neighborhood connectivity between pairs of attributes. The number of strong correlations (Total and Intra-cluster) as well as the pro-inflammatory/regulatory ratio of strong correlations were used for comparative analysis of COVID-19 vs HC and between COVID-19 subgroups (nMV vs MV and Discharge vs Death). Nodes with distinct neighborhood connectivity associates with good (nMV and Discharge) or poor prognosis (MV and Death) are highlighted by continuous or dashed arrows, respectively.

To tailor the multi-functional framework of serum soluble mediators in patients with severe COVID-19 according to disease progression, integrative system networks were constructed (**Figure 2**). Data analysis demonstrated that patients with severe COVID-19 exhibited a more complex and robust network of serum soluble mediators (252 strong correlations) compared to healthy control patients (56 strong correlations). Comparative analysis of the connectivity ratio involving pro-inflammatory/regulatory cytokines further demonstrated a predominance of the pro-inflammatory axis in the COVID-19 group (ratio = 1.9) compared to the HC (ratio = 0.8).

Additional data analysis of the COVID-19 subgroups demonstrated that patients requiring mechanical ventilation (MV) or those progressing to Death displayed a more complex network (362 and 302 strong correlations, respectively) compared to those patients evolving without mechanical ventilation (nMV) or evolving to Discharge (274 and 264, respectively). Moreover, analysis of the connectivity ratio involving pro-inflammatory/regulatory cytokines further demonstrated a predominance of the pro-inflammatory axis at the time of hospital admission (D0) that was associated with better prognosis (nMV or Discharge; ratio = 1.9) in comparison with patients with a worse prognosis (MV or Death; ratio = 1.3 and 1.1, respectively). These apparent controversial quantitative findings were further addressed to identify the qualitative profile, focusing on the specific pro-inflammatory and regulatory mediators involved. The results demonstrated that the proinflammatory and regulatory axis of patients progressing with mechanical ventilation or evolving to death included a higher number of strong correlations involving IFN-γ, IL-1Ra and IL-9 compared to those maintained without mechanical ventilation or with a discharge outcome (“nMV vs MV” and “Discharge vs Death” as follows: IFN-γ = 9 vs 15 and 6 vs 12; IL-1Ra = 9 vs 16 and 8 vs 14; IL-9 = 16 vs 0 and 14 vs 0) (**Figure 2**).

### Multivariate analysis of serum soluble mediators in severe COVID-19 patients on hospital admission

**Figure 3:**
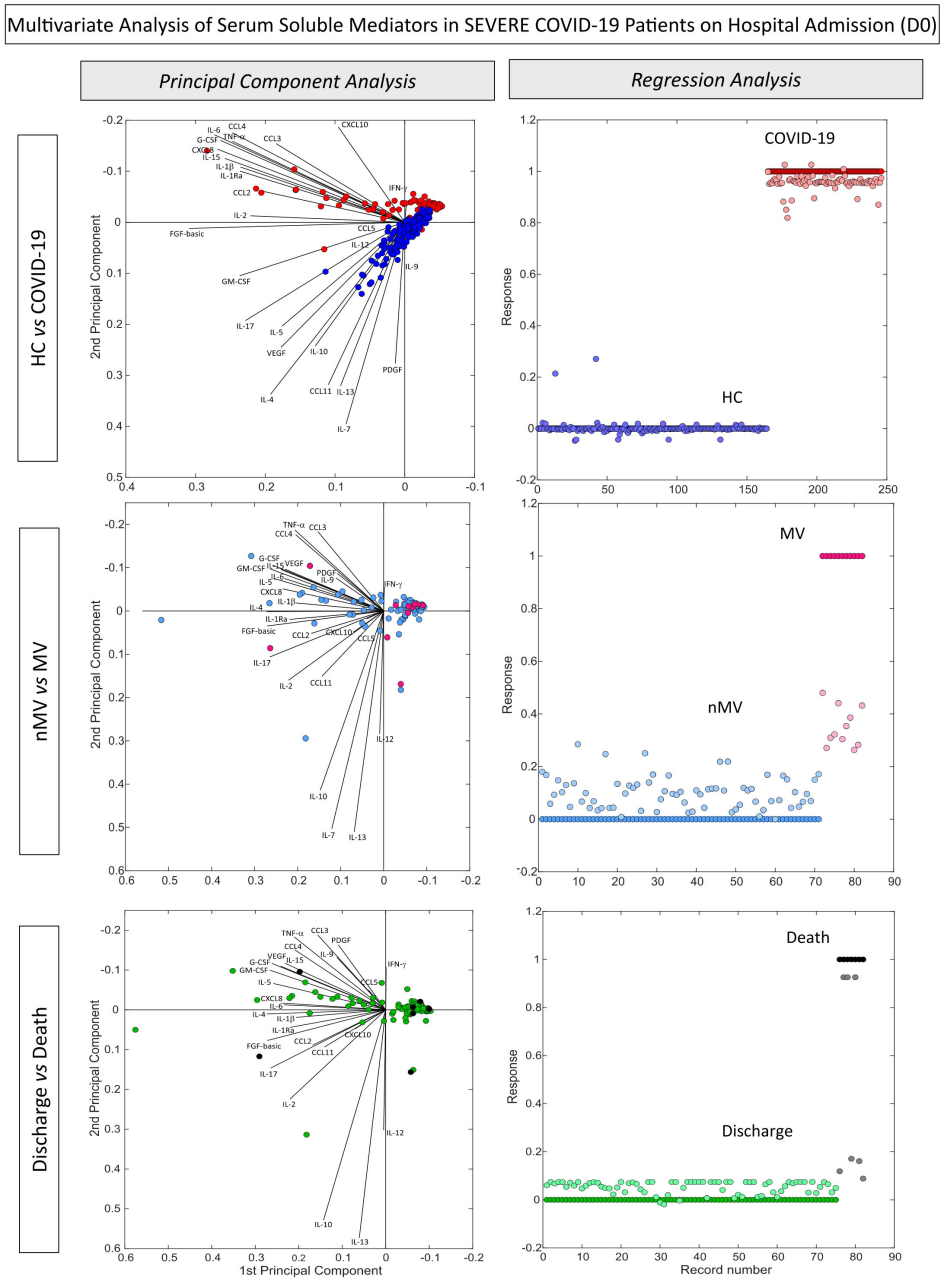
*Multivariate analysis of serum soluble mediators in severe COVID-19 patients according to disease outcome.* Multivariate analysis of serum chemokines, pro-inflammatory cytokines, regulatory cytokines and growth factors were performed to verify the ability of serum soluble mediators to cluster COVID-19 (n=82) apart from and non-infected pre-pandemic Healthy Controls (HC, n=164) as well as subgroups of COVID-19, according to disease outcome, referred to as: Non-Mechanical Ventilation (nMV, n=71) vs Mechanical Ventilation (MV, n=11) and Discharge (n=75) vs Death (n=7). The soluble mediators were measured by Luminex Bio-Plex platform as described in Material and Methods. Principal Component Analysis (PCA) and multivariate regression analysis were carried out by MATLAB software as described in Material and Methods. PCA coordinates (1^st^ and 2^nd^ principal components) were used to compare and visualize the grouping of COVID-19 vs HC, nMV vs MV and Discharge vs Death. The multivariate regression model was trained using regression learner, assigning values of 0 to COVID-19, nMV and Discharge classes and values of 1 to HC, MV and Death classes. The selected model for analysis was obtained after 10 times training based on the highest R-squared.

Multivariate analysis of chemokines, pro-inflammatory cytokines, regulatory cytokines and growth factors were performed using PCA and multivariate regression to verify the ability of serum mediators to cluster COVID-19 apart from non-infected pre-pandemic healthy controls (**Figure 3**). PCA coordinates (1^st^ and 2^nd^ principal components) demonstrated that patients with COVID-19 were grouped into a distinct cluster from HC. The vector analysis indicated that CXCL8, CCL3, CCL4, CCL2, CCL5, CXCL10, IL-1β, IL-6, TNF-α, IFN-γ, IL-15, IL-1Ra, IL-9, FGF-basic, G-CSF and IL-2 were associated with COVID-19, while CCL11, IL-12, IL-4, IL-5, IL-10, IL-13, PDGF, VEGF, GM-CSF and IL-7 related to non-infected controls. Multivariate regression analysis further confirmed the ability of serum mediators to cluster COVID-19 from HC. Conversely, the PCA and multivariate regression outputs did not demonstrate the ability of the serum soluble mediator profile to cluster COVID-19 subgroups according to clinical outcome (**Figure 3**).

### Magnitude of changes in serum soluble mediators on hospital admission of severe COVID-19 patients according to disease outcome

**Figure 4:**
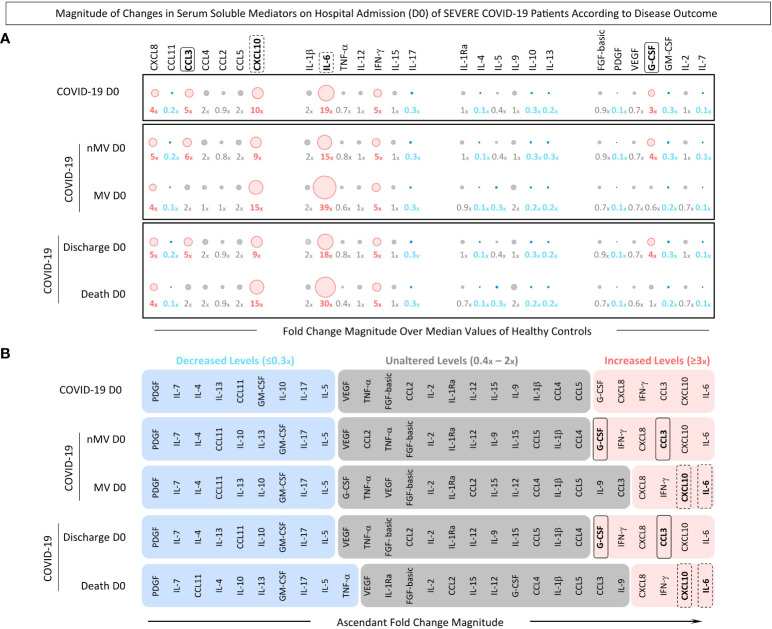
*Magnitude of changes in serum soluble mediators on hospital admission (D0) of severe COVID-19 patients according to disease outcome*. The fold change magnitude of serum chemokines (CXCL8, CCL11, CCL3, CCL4, CCL2, CCL5, CXCL10), pro-inflammatory cytokines (IL-1β, IL-6, TNF-α, IL-12, IFN-γ, IL-15, IL-17), regulatory cytokines (IL-1Ra, IL-4, IL-5, IL-9, IL-10, IL-13), and growth factors (FGF-basic, PDGF, VEGF, G-CSF, GM-CSF, IL-2, and IL-7) were calculated COVID-19 patients (n=82), further categorized into subgroups according to disease outcome, referred to as: Non-Mechanical Ventilation (nMV, n=71) vs Mechanical Ventilation (MV, n=11) and Discharge (n=75) vs Death (n=7). Serum soluble mediators were measured by high-throughput multiplex bead array as described in Material and Methods. The results are presented as the proportion of levels observed for each COVID-19 patient at admission (D0) divided by the median values reported for non-infected pre-pandemic Healthy Controls (HC, n=164). **(A)** Orbital graphs of fold change magnitudes underscore the attributes with decreased (≤0.3x, blue), unaltered (0.4-2x, gray) or increased levels (≥3x, pink) in relation to the median values observed in HC. **(B)** Ascendant signatures of fold change magnitudes were assembled to identify serum soluble mediators of good (nMV and Discharge, continuous rectangles) or poor prognosis (MV and Death, dashed rectangles).

To assess the magnitude of changes in serum levels of soluble mediators in patients with severe COVID-19, the fold change ratios in chemokines, pro-inflammatory cytokines, regulatory cytokines and growth factors were calculated as the proportion between the serum levels observed for each COVID-19 patient at admission (D0) divided by the median values reported for non-infected pre-pandemic Healthy Controls (HC) (**Figure 4**).

Data analysis demonstrated that COVID-19 patients presented a clear pro-inflammatory storm, mediated by IL-6 and CXCL10 with the highest magnitude order (≥19x; ≥10x, respectively). Conversely, an impaired regulatory profile was also observed with decreased levels of IL-4, IL-13 and IL-10 (0.1x, 0.2x and 0.3x, respectively). G-CSF was identified as the single growth factor with increased magnitude in the COVID-19 patients. Further analysis was carried out to evaluate the order of magnitude in COVID-19 patients sub-grouped according to disease outcome: Non-Mechanical Ventilation (nMV) vs Mechanical Ventilation (MV) and Discharge vs Death. The results showed that patients within the nMV and Discharge subgroups presented higher magnitude order orders for CCL3 (6x and 5x, respectively) and G-CSF (4x). Conversely, patients from the MV and Death subgroups displayed a higher magnitude of increase for IL-6 (39x and 30x, respectively) and CXCL10 (15x) (**Figure 4A**).

Ascendant signatures of fold change magnitude were further assembled to underscore the set of soluble mediators with increased magnitude orders capable of identifying subgroups of COVID-19 patients. The results showed that G-CSF and CCL3 were selectively increased in patients with good prognosis (nMV and Discharge). Moreover, although CXCL10 and IL-6 were universally found with increased levels in all COVID-19 subgroups, the magnitude order was higher in MV and Death compared to nMV and Discharge (**Figure 4B**).

### Timeline kinetics of fold changes in serum soluble mediators in severe COVID-19 patients according to disease outcome

**Figure 5:**
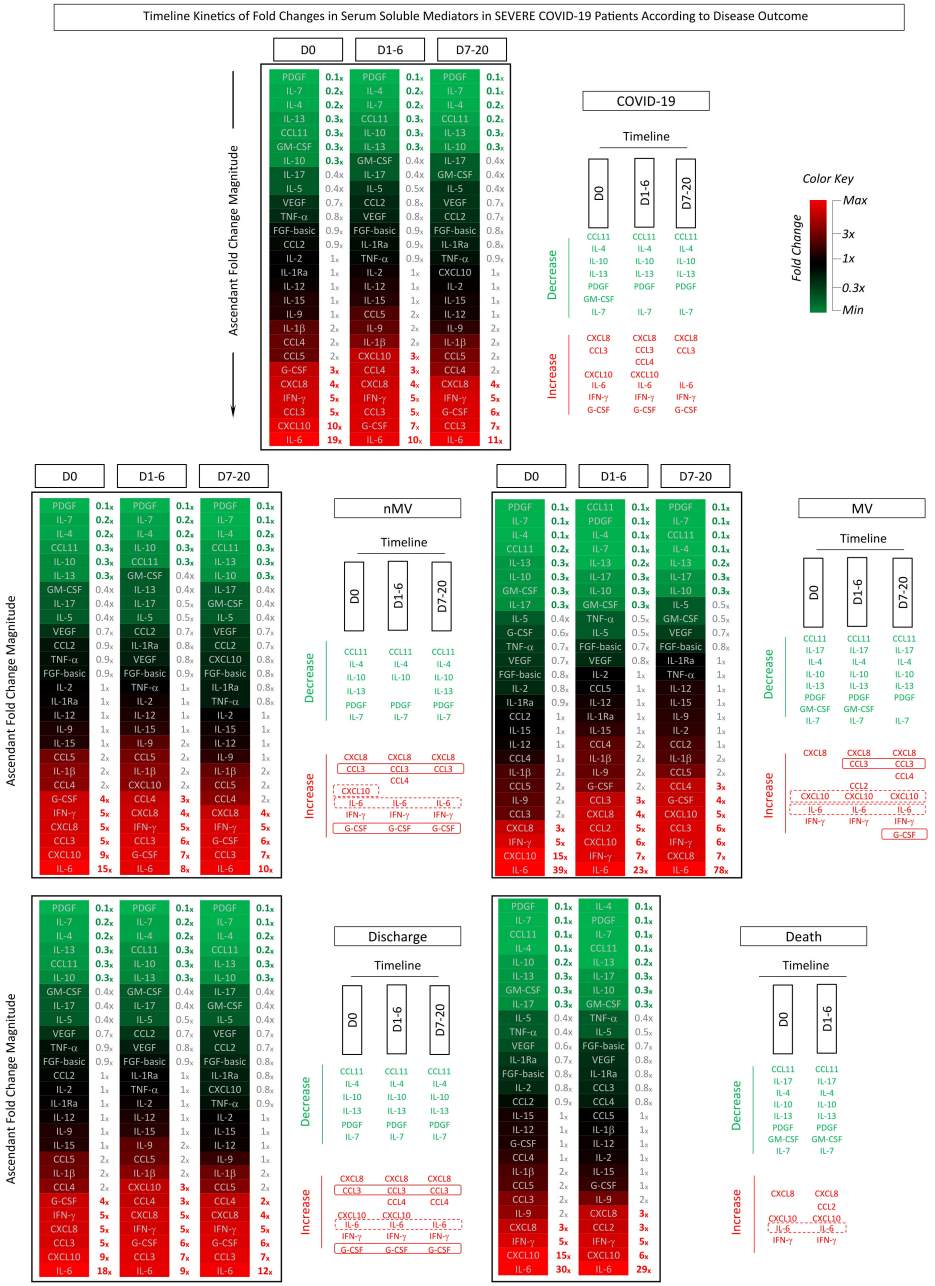
*Timeline kinetics of fold changes in serum soluble mediators in severe COVID-19 patients according to disease outcome*. The fold changes in serum chemokines (CXCL8, CCL11, CCL3, CCL4, CCL2, CCL5, CXCL10), pro-inflammatory cytokines (IL-1β, IL-6, TNF-α, IL-12, IFN-γ, IL-15, IL -17), regulatory cytokines (IL-1Ra, IL-4, IL-5, IL-9, IL-10, IL-13) and growth factors (FGF-basic, PDGF, VEGF, G-CSF, GM- CSF, IL-2, IL-7) were assessed along the kinetic timeline follow-up after admission (D0, D1-6 and D7-20) in COVID-19 patients (n=82), further categorized into subgroups according to disease outcome, referred to as: Non-Mechanical Ventilation (nMV, n=71) vs Mechanical Ventilation (MV, n=11), and Discharge (n=75) vs Death (n=7). Serum soluble mediators were measured by high-throughput multiplex bead array as described in Material and Methods. The results are presented as the proportion of levels observed for each COVID-19 patient along the kinetic timeline follow-up divided by the median values reported for non-infected pre-pandemic Healthy Controls (HC, n=164). Data are presented in heatmap constructs illustrating the ascendant signature of fold change magnitudes along the kinetic timeline. A color key was used to underscore the attributes with decreased (fold change <1x, towards green), unaltered (fold change =1x, black) or increased levels (fold change >1x, towards red. Further analysis was carried out by timeline diagrams to identify common and selective soluble mediators (rectangles) with decreased (≤0.3x, green) or increased (≥3x, red) levels along the kinetic timeline. Serum soluble mediators of good (nMV and Discharge) or poor prognosis (MV and Death) are highlighted by continuous or dashed rectangles, respectively.

To describe a comprehensive kinetic timeline of serum soluble mediators, the fold change magnitude of serum soluble mediators was evaluated in patients with severe COVID-19 at three 3 consecutive timepoints: D0 (n=82), D1-6 (n=84), and D7-20 (n=46) (**Figure 5**).

Data analysis demonstrated an increased order of magnitude (≥3x) for CXCL8, CCL3, IL-6, IFN-γ, G-CSF and decreased order of magnitude (≤0.3x) for CCL11, IL-4, IL-5, IL-10, PDGF and IL-7 in COVID-19 patients throughout the kinetic follow-up. An increased order of magnitude was observed throughout the kinetic timeline for IL-6 (D0 = 19x; D1-6 = 10x; D7-20 = 11x), while a progressive waning of CXCL10 levels was observed from D0 to D7-20 (D0 = 10x; D1-6 = 3x; D7-20 = 1x). Heatmap constructs further illustrated these findings (**Figure 5**).

The dynamic kinetics of serum soluble mediators were further investigated in COVID-19 patients classified according to the disease outcome, referred to as: nMV vs MV and Discharge vs Death (**Figure 5**). Data analysis showed that while patients progressing to MV presented an increasing order of magnitude for IL-6 from D0 to D7-20 (39x to 78x), patients that evolved to the nMV outcome showed a modest decreasing profile at matching timepoints (15x to 10x). Additionally, patients progressing to MV presented elevated orders of magnitude of CXCL10 at all timepoints (D0 = 15x; D1-6 = 6x; D7-20 = 5x), whereas nMV patients only displayed increased CXCL10levels at D0 with a progressive decline to D7-20 (D0 = 9x; D1-6 = 2x; D7-20 = 0.8x). Comparative analysis of COVID-19 patients according to Discharge or Death outcome demonstrated that the latter presented higher IL-6 and CXCL10 levels at corresponding timepoints (**Figure 5**).

Increased CCL3 and G-CSF levels were only observed on admission (D0) and along the kinetic timeline in patients with a favorable outcome (nMV and Discharge), whereas increases in CCL3 and G-CSF were delayed, or even absent, in patients with a poor prognosis (MV and Death) (**Figure 5**).

### Performance of serum soluble mediators to categorize severe COVID-19 patients according to disease outcome

**Figure 6:**
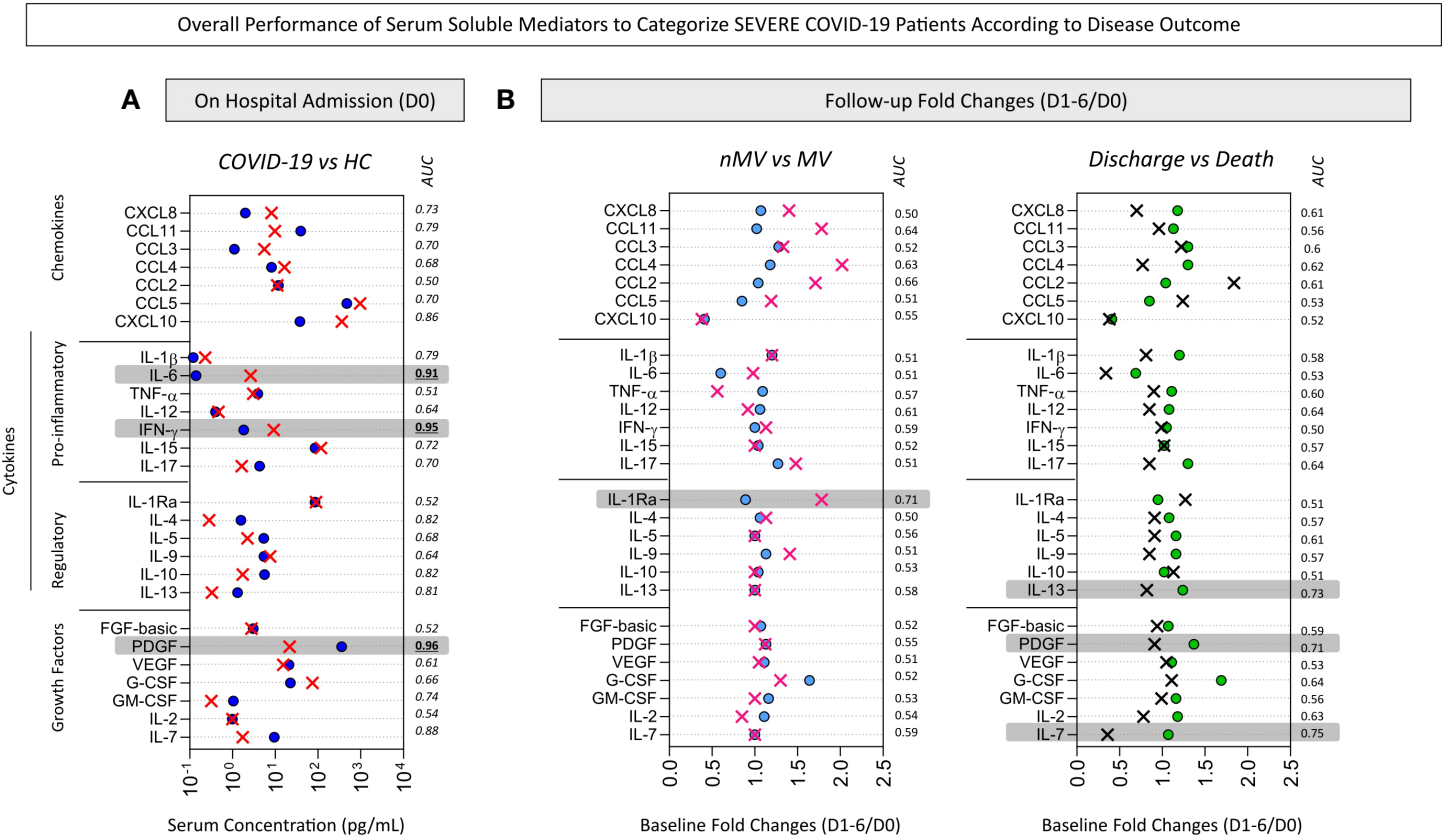
*Overall performance of serum soluble mediators to categorize severe COVID-19 patients according to disease outcome.* The performance of serum chemokines (CXCL8, CCL11, CCL3, CCL4, CCL2, CCL5, CXCL10), pro-inflammatory cytokines (IL-1β, IL-6, TNF-α, IL-12, IFN-γ, IL-15, IL -17), regulatory cytokines (IL-1Ra, IL-4, IL-5, IL-9, IL-10, IL-13) and growth factors (FGF-basic, PDGF, VEGF, G-CSF, GM- CSF, IL-2, IL-7) to categorize patients with COVID-19 were assessed to identify putative immunological parameters for complementary diagnosis and prognosis purposes. Receiver Operating Characteristic (ROC) curve analysis were constructed for each soluble mediator and the area under the ROC curve (AUC) used as global accuracy scores. **(A)** The serum concentration of soluble mediators (pg/mL) quantified upon admission (D0) were employed to classify COVID-19 patients (red X, n=82) from non-infected pre-pandemic Healthy Controls (HC, royal blue circle, n=164) and the soluble mediators with AUC >0.9 (gray background) pre-selected for further analysis. **(B)** The baseline fold change (D1-6/D0) were employed to classify subgroups of COVID-19 patients according to disease outcome, referred to as: Non-Mechanical Ventilation vs Mechanical Ventilation (nMV, light blue circle, n=71; MV, pink X, n=11) and Discharge vs Death (green circle, n=75; black X, n=7). The baseline fold change was calculated for each soluble mediator as the proportion of serum levels observed at D1-6 upon admission divided by the values reported on admission (D0). Soluble mediators with AUC >0.7 (gray background) were pre-selected for further analysis.

To substantiate the use of immunological parameters as complementary records to support COVID-19 diagnosis and prognosis, the applicability of serum soluble mediators as laboratory markers was investigated considering the concentration levels on admission (D0) together with the change in serum levels along the kinetic timeline reported as baseline fold changes at D1-6 according to D0 (D1-6/D0). The performance indices were assessed by ROC curve analysis and the area under the ROC curve (AUC) used as a global accuracy score (**Figure 6**).

**Figure 7:**
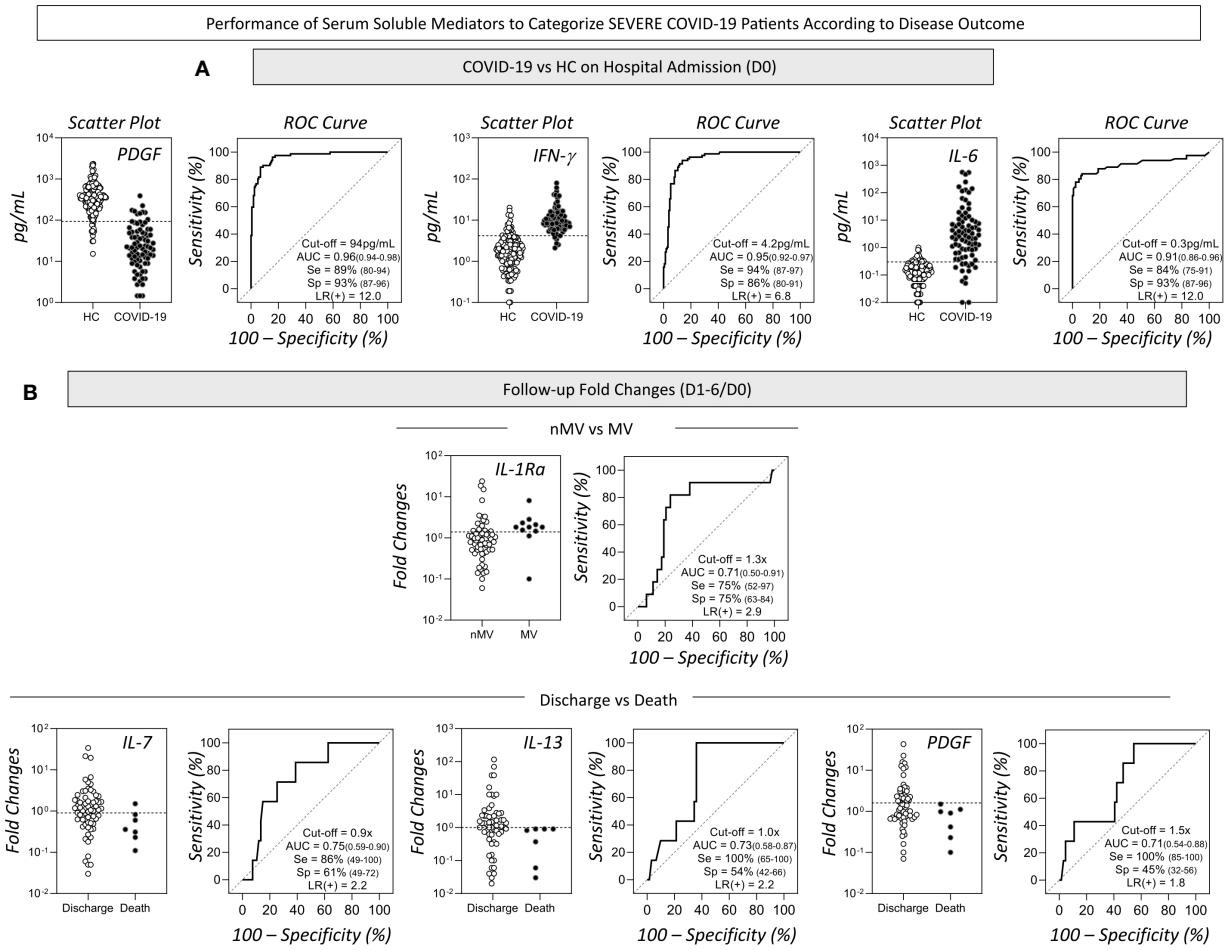
*Performance of serum soluble mediators to categorize severe COVID-19 patients according to disease outcome.* The detailed performance of pre-selected serum soluble mediators to categorize severe COVID-19 patients were assessed employing additional indices to substantiate the use of putative immunological parameters for complementary diagnosis and prognosis purposes. Scatter plot distribution of individual values and Receiver Operating Characteristic (ROC) curve analysis were constructed for each soluble mediator and the performance indices (area under the ROC curve - AUC, Sensitivity-Se, Specificity-Sp and Likelihood ratio - LR) are provided in the figure. **(A)** The serum concentration of PDGF, IFN-γ and IL-6 (pg/mL) quantified upon admission (D0) displayed higher performance to classify COVID-19 patients (black circles, n=82) from non-infected pre-pandemic Healthy Controls (HC, white circle, n=164). Specific cut-offs were defined for each selected soluble mediator as follows: PDGF (94pg/mL); IFN-γ (4.2pg/mL) and IL-6 (0.3pg/mL). **(B)** The baseline fold change (D1-6/D0) of IL-1Ra presented higher performance to classify Non-Mechanical Ventilation (nMV, white circle, n=71) from Mechanical Ventilation (MV, black circle, n=11). IL-7, IL-13 and PDGF showed higher performance to classify Discharge (white circle, n=75) from Death (black circle, n=7). Specific cut-offs were defined for the fold change (D1-6/D0) of each selected soluble mediator as follows: IL-1Ra (1.3x); IL-7 (0.9x) and IL-13 (1.0x) and PDGF (1.5x).

The performance indices of serum soluble mediators assessed on admission (D0) to classify COVID-19 patients and disease outcome are shown in **Figure 6A**. The data demonstrated that PDGF, IFN-γ and IL-6 exhibited elevated global accuracy (AUC>0.9) as reliable soluble mediators to sort COVID-19 patients from HC. To further explore the performance of these pre-selected serum soluble mediators to classify COVID-19 patients, scatter plot distribution and ROC curve analysis were assembled (**Figure 7**). The scatter plot distribution of individual values showed that decreased levels of PDGF (cut-off = 94 pg/mL; Se=89%; Sp=93%) and increased levels of IFN-γ (cut-off=4.2 pg/mL; Se=94%; Sp=86%) and IL-6 (cut-off=0.3 pg/mL; Se=84%; Sp=93%) on hospital admission (D0) were able to correctly differentiate the COVID-19 patients from the healthy control patients (**Figure 7A**). A detailed description of the performance indices of serum soluble mediators measured at baseline (D0) is presented in **Supplementary Table 1**.

The performance of serum soluble mediators reported along the kinetic timeline as baseline fold changes (D1-6/D0) for the classification of COVID-19 subgroups according to disease outcome is shown in **Figure 6B**. The data demonstrated that IL-1Ra presented a high performance (AUC=0.71) to distinguish nMV from MV. Moreover, IL-7, IL-13 and PDGF showed better performance to classify Discharge vs Death (AUC=0.75, 0.73 and 0.71, respectively). The aforementioned soluble mediators were pre-selected for further analysis (**Figure 7B**). The scatter plot distribution of individual values showed that increased fold changes in IL-1Ra levels (cut-off=1.3x; Se and Sp=75%) were able to correctly distinguish MV from nMV. Additionally, decreased fold changes in IL-7 (cut-off=0.9x; Se=86%; Sp=61%), IL-13 (cut-off=1.0x; Se=100%; Sp=54%), and PDGF (cut-off=1.3x; Se=100%; Sp=45%), were associated with the Death outcome (**Figure 7B**). A detailed description of the performance indices of changes in serum soluble mediator levels along the kinetic timeline (D1-6/D0) is presented in **Supplementary Table 2**.

## Discussion

The present study was designed as an exploratory investigation employing distinct models of descriptive analysis to characterize the panoramic profile of serum soluble mediators in severe COVID-19 patients according to disease outcome.

Our results demonstrated that overall, the COVID-19 group exhibited a massive storm of soluble mediators, especially chemokines and pro-inflammatory cytokines, in comparison to the healthy control group. Several studies have already demonstrated that COVID-19 elicits a massive release of various immunological mediators, comprising high levels of chemokines and pro-inflammatory cytokines (11–13). It is strongly believed that pro-inflammatory cytokine hyper-inductions is one of the key aspects of the current SARS-CoV-2 pandemic, and one of the factors contributing to the mortality observed with COVID-19 for a subgroup of patients (14, 15). It has been proposed that some soluble mediators were predictive of subsequent clinical progression, and that monitoring these biological markers on hospital admission and during follow-up could prove useful in identifying patients at risk of disease complications and progression to death (16). In this sense, the results obtained in the present investigation demonstrated that distinct patterns of serum soluble mediators were visualized at the time of hospital admission (D0) and during the progression to disease outcomes. While patients evolving to nMV and Discharge presented higher CCL3 and G-CSF levels, patients progressing to MV and Death exhibited higher CXCL10 and IL-6 levels, together with lower IL-10 levels.

Previous studies proposed that monocytes and lung macrophage subsets play a key role in pathological inflammation in COVID-19 patients, mediating the extensive lung and vascular tissue damage (17, 18). Macrophages are pivotal cells, key components of the innate immune system involved in the detection, phagocytosis, and resolution of many inflammatory disorders. In addition, these cells may also present antigens to T-cells and initiate inflammation by releasing key cytokines, including a range of chemokines (e.g. CXCL10 and CXCL11) and pro-inflammatory molecules (e.g. TNF and IL-1β) with anti-microbial functions, but in some cases the uncontrolled cytokine storm may aggravate tissue damage (19). Studies focusing on single-cell RNA sequencing of COVID-19 patient bronchoalveolar lavage fluid pointed out that macrophage subsets producing CCL2 and CXCL10 are abundant in the bronchoalveolar lavage fluid from severe COVID-9 patients and might be potential mediators in the COVID-19 disease (20). Although the present investigation did not analyze cellular components, based on our findings, we hypothesize that the monocyte/macrophage activation during COVID-19 culminates in increased CXCL10 levels that ultimately contribute to the non-favorable clinical outcomes (MV and Death), signalizing the importance of scaling up chemokine-orchestrated immunity.

Overall, our findings are in general agreement with previous studies, however, there are a number of controversial results in comparison to other reports. The marked increase in CXCL10 and IL-6 corroborates previous studies describing the immune response in COVID-19 patients associated with disease severity and progression (3, 11–13, 21–24). Conversely, the association of G-CSF with a favorable prognosis was not aligned with previous reports demonstrating that increased G-CSF levels were linked with COVID-19 severity (11). In agreement with our findings associating G-CSF levels with a good prognosis, previous studies demonstrated that the administration of G-CSF increased the number of lymphocytes, T-cells, thus improving the clinical symptoms in severe COVID-19 patients (25). As G-CSF is a potent hematopoiesis stimulating factor (26), higher G-CSF levels in early-onset COVID-19 patients may contribute to protecting the host against infection, preventing the development of lymphopenia observed in severe and fatal COVID-19 cases during the first days from illness onset (27–31). The apparent conflicting data in the literature regarding the role of G-CSF in COVID-19 outcome may relate to the timing of G-CSF measurement that is relevant to identify its association with a protective or deleterious role. Additional studies with larger numbers of patients are required to better understand the connection between G-CSF and disease progression in COVID-19 patients. Additionally, our results have demonstrated a significant decrease of PDGF levels in COVID-19 patients as compared to healthy controls. Previous studies have reported higher levels of PDGF-AA and PDGF-BB in COVID-19 patients (32). These apparently controversial finding may reflect intrinsic clinical features of the study population. In that study, the authors mentioned that the cohort exhibited relatively normal platelet counts and that all COVID-19 patients were received standard thromboprophylaxis treatment (32). Moreover, while they have measured the PDGF-AA and PDGF-BB levels in plasma samples, in our study we have measured the PDGF levels in serum samples. Several studies have highlighted that many growth factors can accumulate in platelet α-granules, a fact that accounts for the major differences observed between measurements in plasma vs serum (33, 34). In this sense, the use of a transformation factors to normalize the PDGF levels is recommended to allow accurate comparisons between different studies. In the present study, we did not assess the hematological parameters of patients and controls at the time of sample collection for soluble mediator measurements and therefore these correction factors could not be applied. Further investigations considering these corrections factors are relevant to normalize data and make them comparable between distinct studies.

Our data have shown that IL-10 levels were decreased in COVID-19 patients and even lower in patients progressing to death. A metanalysis carried out by Hu and colleagues (35) have reported that while higher levels of IL-10 were higher in the severe or death COVID-19 subgroups. However, the authors mentioned that the levels of IL-10 correlated with gender, being significantly higher in males than in females and also different in COVID-19 patients from distinct geographical regions, been a potential biomarker to predict the risk of mortality in Asian patients, but not in European patients (35). We hypothesize that these particularities such and gender distribution and/or distinct genetic background according to distinct geographical regions may explain the apparently contradictory findings.

It has been shown that SARS‐CoV‐2 spike boosts VEGF levels. High levels of VEGF were observed in previous studies and anti-VEDF therapy proposed to control COVID-19 in critically ill patients (36), especially those with gastrointestinal symptoms correlated with intestinal edema and disease progression (37). Conversely, our data have demonstrated that VEGF levels were lower in COVID-19 patients as compared to healthy controls. To be noticed, VEGF levels in blood of the animal model is not increased at the initial stages and significantly boosted upon continuous treatment with spike RBD, suggesting that kinetic differences could be observed from a local to systemic spread (37). Therefore, difference in the early and late timeline kinetics of VEGF should be considered as a plausible explanation for the differences observed between clinical studies.

All in all, we believe that the apparently conflicting results reported in the literature may be the result of distinct aspects of the study population, including co-morbidities and other host-related factors (age, sex) as well as the timeline between infection and hospital admission (38–42). It is possible that the analysis of the immune response, considering the kinetic timeline from symptoms onset or from the time of hospital admission, may lead to distinct findings.

It is well known that anti-inflammatory therapy in place for severe cases of COVID-19 might affect the chemokines/cytokines/growth factors levels. In the present study, all COVID-19 patients received a combined corticosteroids/antibiotic/heparin therapy regardless the evolution to mechanical ventilation or disease outcome to death. Therefore, the differences observed in the levels of serum soluble mediators between COVID-19 subgroups may not represent an impact of therapy as a confounding variable.

Aiming to further characterize the pro-inflammatory storm observed in severe COVID-19 patients, we assessed the magnitude of changes in the levels of serum soluble mediators in severe COVID-19 patients along the kinetic timeline in comparison to the median values observed in serum samples from healthy controls. Our findings identified and further pointed out that IL-6 and CXCL10 had the highest order of magnitude in patients with poor prognoses (MV and Death), while G-CSF and CCL3 were selectively increased in patients with a good prognosis.

Despite the substantial number of studies involving soluble mediators in COVID-19 patients, predictive biomarkers are critically lacking (43). For this purpose, we performed a detailed analysis of serum soluble mediators and constructed integrative system networks. An unbalanced integrative network with a predominance of the pro-inflammatory axis on hospital admission (D0) was associated with better prognosis while qualitative analysis revealed that patients with worse prognosis (MV and Death) had a higher number of strong correlations involving IFN-γ, IL-1Ra and IL-9. This mixed pro-inflammatory/regulatory profile may suggest that these patients (MV and Death) already presented a longer disease duration and had a later hospital admission in comparison to patients who had more favorable disease outcomes (nMV and Discharge).

Taken together, these findings reinforce the well-known pro-inflammatory storm observed in severe COVID-19 patients and further demonstrate unbalanced integrative networks with the simultaneous participation of IFN-γ, IL-1Ra and IL-9 on hospital admission. Furthermore, increased orders of magnitude and disturbed kinetics of serum soluble mediators were associated with poor disease outcomes in severe COVID-19patients.

With the objective of providing useful laboratory biomarkers with predictive values for clinical application, we applied robust statistical methods to identify a set of serum soluble mediators with a good performance to classify distinct COVID-19 outcomes. The ROC curves assembled highlighted that PDGF, IFN-γ and IL-6 presented substantial global accuracy to categorize COVID-19 and HC patients. Furthermore, IL-1Ra, IL-13, PDGF and IL-7 were also identified as promising molecules to distinguish COVID-19 patients progressing to a worse prognosis (MV or Death). Overall, these results demonstrate the ability of a selected set of systemic mediators (IL-6, IFN-γ, IL-1Ra, IL-13, PDGF and IL-7) as putative laboratory markers that are applicable as complementary records in the clinical management of severe COVID-19 patients.

The present study has some limitations. The small number of patients enrolled re-enforces the need to further validate our findings. Particularly, the low number deceased patients as compared to those progressing to discharge may impact the strength of our conclusions. The study was carried out during the circulation of the B.1.1.28 and B.1.1.33 SARS-CoV-2 strains and other variants may lead to distinct immunological profiles. Loss to follow-up did not allow the prospective longitudinal analysis of all patients enrolled on hospital admission. The observational design with multiple comparisons without corrections by co-morbidities for confounding variables also constituted a limitation. Additional studies are required to access the impact of sex and aging on these immunological findings.

In conclusion, the main findings of this study pointed out that in addition to the well-known pro-inflammatory storm, disturbed integrative networks and increased orders of magnitude along the kinetic timeline of serum soluble mediators (specially CXCL10, IL-6, CCL3 and G-CSF) were associated with distinct disease outcomes in severe COVID-19 patients. Moreover, a selected set of serum soluble mediators (IL-6, IFN-γ, IL-1Ra, IL-13, PDGF and IL-7) were pointed out as promising biomarkers for the clinical management of severe COVID-19 patients.

## Data availability statement

The original contributions presented in the study are included in the article/**Supplementary Material** Further inquiries can be directed to the corresponding authors.

## Ethics statement

The studies involving human participants were reviewed and approved by National Commission for Ethics in Research in Brazil (CONEP). The patients/participants provided their written informed consent to participate in this study.

## Author contributions

Study Design: GJ-S, HS, VV, OM-F, and LM. Advisory Committee: PK, CG, ON, CA, LE, AN, AS, PA, and WF. Funding Acquisition: CG, LE, LN, OM-F, and LM. Sample collection, experimental procedures and data acquisition: GJ-S, HS, PK, CG, EG, MC, FM, AC-A, and VP-M. Data Analysis: GJ-S, HS, LA, MG, PB, JB-D-S, AC-A, OM-F, and LM. Writing and reviewing the manuscript: GJ-S, HS, ON, CA, LE, MG, PB, AT-C, VV, OM-F, and LM. All authors contributed to the article and approved the submitted version.

## Funding

This study was supported by the Conselho Nacional de Desenvolvimento Científico e Tecnológico - CNPq. Funding was also provided by the ArboControl Project (TED 74/2016). In addition, this project was funded by Universidade de Brasília, Fundo Covid-19 UnB em Ação: Ações emergenciais para combate à Covid-19 e mitigação das consequências da pandemia, project “Eficácia de um protocolo de testagem RT-PCR para SARS-CoV-2 sobre a preservação da força de trabalho em saúde, durante a pandemia COVID-19 no Brasil: ensaio clínico randomizado, de grupos paralelos".

## Acknowledgments

This study was performed by students and professors enrolled in Post-graduate Programs: Programa de Pós-Graduação em Ciências Médicas da Universidade de Brasília – UnB, supported by Coordenação de Aperfeiçoamento de Pessoal de Nível Superior (CAPES). The authors thank the Program for Technological Development in Tools for Health-RPTFIOCRUZ for use of the flow cytometry facilities. The authors also express thanks to Dayane Andriotti Otta for technical support. ON, AN, PA, AT-C, and OM-F received PQ fellowships from CNPq. AT-C and OM-F are research fellows from FAPEAM (PVN-II, PRÓ-ESTADO Program #005/2019). OAMF and ATC are also grateful to the FAPEAM (PVN-II, PRÓ-ESTADO Program #005/2019) for the research fellowship program.

## Conflict of interest

LM received personal or institutional support from Abbvie, Janssen, Pfizer, Boehringer-Ingelheim, Sandoz and Roche; has delivered speeches at events and sponsored by Abbvie, Boheringer- Ingelheim, Janssen, Pfizer, Roche, Sandoz, Lilly and UCB.

The remaining authors declare that the research was conducted in the absence of any commercial or financial relationships that could be construed as a potential conflict of interest.

## Publisher’s note

All claims expressed in this article are solely those of the authors and do not necessarily represent those of their affiliated organizations, or those of the publisher, the editors and the reviewers. Any product that may be evaluated in this article, or claim that may be made by its manufacturer, is not guaranteed or endorsed by the publisher.

## References

[1] World Health Organization. WHO coronavirus (COVID-19) dashboard data. Available at: https://covid19.who.int/.2022 (AccessedJune 28, 2022).

[2] Beeching NJ Fletcher TE Fowler RE. Coronavirus disease 2019 (COVID-19) - symptoms, diagnosis and treatment(2022) (Accessed June 28, 2022).

[3] Darif D Hammi I Kihel A El Idrissi Saik I Guessous F Akarid K. The pro-inflammatory cytokines in COVID-19 pathogenesis: What goes wrong? Microb Pathogen (2021) 153:104799. doi: 10.1016/j.micpath.2021.104799 33609650 PMC7889464

[4] Asakura H Ogawa H. COVID-19-associated coagulopathy and disseminated intravascular coagulation. Int J Hematol (2021) 113(1):45–57. doi: 10.1007/s12185-020-03029-y 33161508 PMC7648664

[5] Chen R Lan Z Ye J Pang L Liu Y Wu W. Cytokine storm: The primary determinant for the pathophysiological evolution of COVID-19 deterioration. Front Immunol (2021) 12. doi: 10.3389/fimmu.2021.589095 PMC8115911 33995341

[6] Yan Q Li P Ye X Huang X Feng B Ji T. Longitudinal peripheral blood transcriptional analysis reveals molecular signatures of disease progression in COVID-19 patients. J Immunol (2021) 206(9):2146–59. doi: 10.4049/jimmunol.2001325 33846224

[7] Zanza C Romenskaya T Manetti AC Franceschi F La Russa R Bertozzi G. Cytokine storm in COVID-19: Immunopathogenesis and therapy. Med (B Aires) (2022) 58(2):144. doi: 10.3390/medicina58020144 PMC8876409 35208467

[8] Lentz S Roginski MA Montrief T Ramzy M Gottlieb M Long B. Initial emergency department mechanical ventilation strategies for COVID-19 hypoxemic respiratory failure and ARDS. Am J Emergency Med (2020) 38(10):2194–202. doi: 10.1016/j.ajem.2020.06.082 PMC7335247 33071092

[9] Samprathi M Jayashree M. Biomarkers in COVID-19: An up-To-Date review. Front Pediatr (2021) 8. doi: 10.3389/fped.2020.607647 PMC8042162 33859967

[10] Kurizky P Nóbrega OT Soares AADSM Aires RB De Albuquerque CP Nicola AM. Molecular and cellular biomarkers of COVID-19 prognosis: Protocol for the prospective cohort TARGET study. JMIR Res Protoc (2021) 10(3):e24211. doi: 10.2196/24211 33661132 PMC7935398

[11] Guo J Wang S Xia H Shi D Chen Y Zheng S. Cytokine signature associated with disease severity in COVID-19. Front Immunol (2021) 12. doi: 10.3389/fimmu.2021.681516 PMC8418386 34489933

[12] Ling L Chen Z Lui G Wong CK Wong WT Ng RWY. Longitudinal cytokine profile in patients with mild to critical COVID-19. Front Immunol (2021) 12. doi: 10.3389/fimmu.2021.763292 PMC8685399 34938289

[13] Ghazavi A Ganji A Keshavarzian N Rabiemajd S Mosayebi G. Cytokine profile and disease severity in patients with COVID-19. Cytokine (2021) 137:155323. doi: 10.1016/j.cyto.2020.155323 33045526 PMC7524708

[14] Olbei M Hautefort I Modos D Treveil A Poletti M Gul L. SARS-CoV-2 causes a different cytokine response compared to other cytokine storm-causing respiratory viruses in severely ill patients. Front Immunol (2021) 12. doi: 10.3389/fimmu.2021.629193 PMC7956943 33732251

[15] Channappanavar R Perlman S. Pathogenic human coronavirus infections: causes and consequences of cytokine storm and immunopathology. Semin Immunopathol (2017) 39(5):529–39. doi: 10.1007/s00281-017-0629-x PMC7079893 28466096

[16] Lucas C Wong P Klein J Castro TBR Silva J Sundaram M. Longitudinal analyses reveal immunological misfiring in severe COVID-19. Nature (2020) 584(7821):463–9. doi: 10.1038/s41586-020-2588-y PMC7477538 32717743

[17] Zhang F Mears JR Shakib L Beynor JI Shanaj S Korsunsky I. IFN-γ and TNF-α drive a CXCL10+ CCL2+ macrophage phenotype expanded in severe COVID-19 lungs and inflammatory diseases with tissue inflammation. Genome Med (2021) 13(1):64. doi: 10.1186/s13073-021-00881-3 33879239 PMC8057009

[18] Merad M Martin JC. Pathological inflammation in patients with COVID-19: a key role for monocytes and macrophages. Nat Rev Immunol (2020) 20(6):355–62. doi: 10.1038/s41577-020-0331-4 PMC7201395 32376901

[19] Ma WT Gao F Gu K Chen DK. The role of monocytes and macrophages in autoimmune diseases: A comprehensive review. Front Immunol (2019) 10. doi: 10.3389/fimmu.2019.01140 PMC6543461 31178867

[20] Liao M Liu Y Yuan J Wen Y Xu G Zhao J. Single-cell landscape of bronchoalveolar immune cells in patients with COVID-19. Nat Med (2020) 26(6):842–4. doi: 10.1038/s41591-020-0901-9 32398875

[21] Pons MJ Ymaña B Mayanga-Herrera A Sáenz Y Alvarez-Erviti L Tapia-Rojas S. Cytokine profiles associated with worse prognosis in a hospitalized Peruvian COVID-19 cohort. Front Immunol (2021) 12. doi: 10.3389/fimmu.2021.700921 PMC8440968 34539631

[22] Coomes EA Haghbayan H. Interleukin-6 in covid-19: A systematic review and meta-analysis. Rev Med Virol (2020) 30(6):1–9. doi: 10.1002/rmv.2141 PMC7460877 32845568

[23] Udomsinprasert W Jittikoon J Sangroongruangsri S Chaikledkaew U. Circulating levels of interleukin-6 and interleukin-10, but not tumor necrosis factor-alpha, as potential biomarkers of severity and mortality for COVID-19: Systematic review with meta-analysis. J Clin Immunol (2021) 41(1):11–22. doi: 10.1007/s10875-020-00899-z 33128665 PMC7602765

[24] Mojtabavi H Saghazadeh A Rezaei N. Interleukin-6 and severe COVID-19: a systematic review and meta-analysis. Eur Cytokine Netw (2020) 31(2):44–9. doi: 10.1684/ecn.2020.0448 PMC7530350 32933891

[25] Chen GB Lin JT Zhang Z Liu L. Effect of recombinant human granulocyte colony-stimulating factor on lymphocyte subsets in patients with COVID-19. Infect Dis (2020) 52(10):759–61. doi: 10.1080/23744235.2020.1790031 32643997

[26] Mu X Liu K Li H Wang FS Xu R. Granulocyte-macrophage colony-stimulating factor: an immunotarget for sepsis and COVID-19. Cell Mol Immunol (2021) 18(8):2057–8. doi: 10.1038/s41423-021-00719-3 PMC8287545 34282298

[27] Melo AKG Milby KM Caparroz ALMA Pinto ACPN Santos RRP Rocha AP. Biomarkers of cytokine storm as red flags for severe and fatal COVID-19 cases: A living systematic review and meta-analysis. PloS One (2021) 16(6):e0253894. doi: 10.1371/journal.pone.0253894 34185801 PMC8241122

[28] Panda S Nanda R Tripathy P Mangaraj M. Immuno-inflammatory predictors of disease severity in COVID-19: A systematic review and meta-analysis. J Family Med Primary Care (2021) 10(3):1102. doi: 10.4103/jfmpc.jfmpc_2196_20 34041137 PMC8140259

[29] Moutchia J Pokharel P Kerri A McGaw K Uchai S Nji M. Clinical laboratory parameters associated with severe or critical novel coronavirus disease 2019 (COVID-19): A systematic review and meta-analysis. PloS One (2020) 15(10):e0239802. doi: 10.1371/journal.pone.0239802 33002041 PMC7529271

[30] Cao X. COVID-19: immunopathology and its implications for therapy. Nat Rev Immunol (2020) 20(5):269–70. doi: 10.1038/s41577-020-0308-3 PMC7143200 32273594

[31] Zheng M Gao Y Wang G Song G Liu S Sun D. Functional exhaustion of antiviral lymphocytes in COVID-19 patients. Cell Mol Immunol (2020) 17(5):533–5. doi: 10.1038/s41423-020-0402-2 PMC7091858 32203188

[32] Petrey AC Qeadan F Middleton EA Pinchuk IV Campbell RA Beswick EJ. Cytokine release syndrome in COVID-19: Innate immune, vascular, and platelet pathogenic factors differ in severity of disease and sex. J Leukoc Biol (2021) 109(1):55–66. doi: 10.1002/JLB.3COVA0820-410RRR 32930456 PMC7902354

[33] Italiano JE Jr Richardson JL Patel-Hett S Battinelli E Zaslavsky A Short S. Angiogenesis is regulated by a novel mechanism: pro- and antiangiogenic proteins are organized into separate platelet alpha granules and differentially released. Blood (2008) 111(3):1227–33. doi: 10.1182/blood-2007-09-113837 PMC2214735 17962514

[34] Klement GL Yip TT Cassiola F Kikuchi L Cervi D Podust V. Platelets actively sequester angiogenesis regulators. Blood (2009) 113(12):2835–42. doi: 10.1182/blood-2008-06-159541 PMC2661866 19036702

[35] Hu H Pan H Li R He K Zhang H Liu L. Increased circulating cytokines have a role in COVID-19 severity and death with a more pronounced effect in males: A systematic review and meta-analysis. Front Pharmacol (2022) 13:802228. doi: 10.3389/fphar.2022.802228 35237162 PMC8883392

[36] Sahebnasagh A Nabavi SM Kashani HRK Abdollahian S Habtemariam S Rezabakhsh A. Anti-VEGF agents: As appealing targets in the setting of COVID-19 treatment in critically ill patients. Int Immunopharmacol (2021) 101(Pt B):108257. doi: 10.1016/j.intimp.2021.108257 34673299 PMC8519896

[37] Zeng FM Li YW Deng ZH He JZ Li W Wang L. SARS-CoV-2 spike spurs intestinal inflammation *via* VEGF production in enterocytes. EMBO Mol Med (2022) 14(5):e14844. doi: 10.15252/emmm.202114844 35362189 PMC9081906

[38] Petrilli CM Jones SA Yang J Rajagopalan H O’Donnell L Chernyak Y. Factors associated with hospital admission and critical illness among 5279 people with coronavirus disease 2019 in new York city: prospective cohort study. BMJ (2020) 369:m1966. doi: 10.1136/bmj.m1966 PMC7243801 32444366

[39] Cummings MJ Baldwin MR Abrams D Jacobson SD Meyer BJ Balough EM. Epidemiology, clinical course, and outcomes of critically ill adults with COVID-19 in new York city: a prospective cohort study. Lancet (2020) 395(10239):1763–70. doi: 10.1016/S0140-6736(20)31189-2 PMC7237188 32442528

[40] Chen Y Klein SL Garibaldi BT Li H Wu C Osevala NM. Aging in COVID-19: Vulnerability, immunity and intervention. Ageing Res Rev (2021) 65:101205. doi: 10.1016/j.arr.2020.101205 33137510 PMC7604159

[41] Garibaldi BT Fiksel J Muschelli J Robinson ML Rouhizadeh M Perin J. Patient trajectories among persons hospitalized for COVID-19. Ann Internal Med (2021) 174(1):33–41. doi: 10.7326/M20-3905 32960645 PMC7530643

[42] Wu Z McGoogan JM. Characteristics of and important lessons from the coronavirus disease 2019 (COVID-19) outbreak in China. JAMA (2020) 323(13):1239. doi: 10.1001/jama.2020.2648 32091533

[43] del Valle DM Kim-Schulze S Huang HH Beckmann ND Nirenberg S Wang B. An inflammatory cytokine signature predicts COVID-19 severity and survival. Nat Med (2020) 26(10):1636–43. doi: 10.1038/s41591-020-1051-9 PMC7869028 32839624

