# Supplementary material for: Unbalanced networks and disturbed kinetics of serum soluble mediators associated with distinct disease outcomes in severe COVID-19 patients
Source: Front Immunol. 2022 Nov 14;13:1004023. doi: 10.3389/fimmu.2022.1004023 (PMC9701840; doi:10.3389/fimmu.2022.1004023)
Supplement: Supplementary file 1 [file Table_1.docx]

*Supplementary Table 1.* Performance indices of serum soluble mediators measured at admission (D0) to classify patients with SEVERE COVID-19 according to disease outcome

| COVID-19 vs HC | | | | | | | |  | nVM vs MV | | | | | | | |  | Discharge vs Death | | | | | | | |
| --- | --- | --- | --- | --- | --- | --- | --- | --- | --- | --- | --- | --- | --- | --- | --- | --- | --- | --- | --- | --- | --- | --- | --- | --- | --- |
| Parameters |  | Cut-off |  | AUC |  | Se | Sp |  | Parameters |  | Cut-off |  | AUC |  | Se | Sp |  | Parameters |  | Cut-off |  | AUC |  | Se | Sp |
| **PDGF** |  | **93.7** |  | **0.96** |  | **89** | **93** |  | CXCL10 |  | 993.1 |  | 0.65 |  | 82 | 46 |  | CCL11 |  | 1.6 |  | 0.65 |  | 88 | 43 |
| **IFN-γ** |  | **4.2** |  | **0.95** |  | **94** | **86** |  | CCL11 |  | 1.6 |  | 0.61 |  | 87 | 36 |  | IL-12 |  | 0.4 |  | 0.64 |  | 39 | 86 |
| **IL-6** |  | **0.3** |  | **0.91** |  | **84** | **93** |  | IL-6 |  | 2.4 |  | 0.62 |  | 52 | 82 |  | CXCL10 |  | 1288.3 |  | 0.64 |  | 89 | 43 |
| IL-7 |  | 4.5 |  | 0.88 |  | 79 | 84 |  | IL-15 |  | 90.4 |  | 0.61 |  | 73 | 60 |  | IL-10 |  | 1.3 |  | 0.6 |  | 77 | 57 |
| CXCL10 |  | 129.7 |  | 0.86 |  | 78 | 95 |  | CCL2 |  | 3.4 |  | 0.61 |  | 23 | 100 |  | IL-6 |  | 115.2 |  | 0.57 |  | 93 | 29 |
| IL-10 |  | 2.3 |  | 0.82 |  | 72 | 88 |  | IL-10 |  | 1.3 |  | 0.59 |  | 76 | 46 |  | IFN-γ |  | 16.7 |  | 0.57 |  | 84 | 43 |
| IL-4 |  | 0.6 |  | 0.82 |  | 68 | 93 |  | GM-CSF |  | 0.4 |  | 0.58 |  | 45 | 73 |  | IL-1Ra |  | 78.0 |  | 0.56 |  | 58 | 71 |
| IL-13 |  | 0.6 |  | 0.81 |  | 74 | 82 |  | IFN-γ |  | 8.1 |  | 0.58 |  | 38 | 82 |  | GM-CSF |  | 0.3 |  | 0.56 |  | 51 | 71 |
| IL-1β |  | 0.1 |  | 0.79 |  | 99 | 42 |  | G-CSF |  | 16.0 |  | 0.58 |  | 71 | 60 |  | IL-9 |  | 13.0 |  | 0.55 |  | 73 | 57 |
| CCL11 |  | 13.0 |  | 0.79 |  | 56 | 91 |  | IL-12 |  | 0.4 |  | 0.57 |  | 37 | 82 |  | CCL2 |  | 3.4 |  | 0.55 |  | 22 | 100 |
| GM-CSF |  | 0.7 |  | 0.74 |  | 68 | 100 |  | FGF-basic |  | 2.9 |  | 0.57 |  | 45 | 82 |  | IL-15 |  | 90.4 |  | 0.55 |  | 70 | 50 |
| CXCL8 |  | 4.0 |  | 0.73 |  | 63 | 79 |  | IL-1β |  | 0.2 |  | 0.56 |  | 49 | 73 |  | VEGF |  | 12.7 |  | 0.54 |  | 65 | 57 |
| IL-15 |  | 101.4 |  | 0.72 |  | 64 | 84 |  | IL-4 |  | 0.2 |  | 0.56 |  | 58 | 64 |  | TNF-α |  | 1.6 |  | 0.54 |  | 72 | 57 |
| CCL5 |  | 699.8 |  | 0.7 |  | 65 | 79 |  | IL-2 |  | 0.8 |  | 0.56 |  | 55 | 64 |  | IL-4 |  | 0.2 |  | 0.54 |  | 60 | 71 |
| IL-17 |  | 1.7 |  | 0.7 |  | 52 | 90 |  | CCL4 |  | 18.2 |  | 0.55 |  | 51 | 73 |  | CXCL8 |  | 1.9 |  | 0.53 |  | 25 | 100 |
| CCL3 |  | 4.1 |  | 0.7 |  | 53 | 94 |  | TNF-α |  | 3.5 |  | 0.55 |  | 44 | 73 |  | FGF-basic |  | 2.7 |  | 0.53 |  | 53 | 71 |
| IL-5 |  | 2.4 |  | 0.68 |  | 61 | 82 |  | IL-5 |  | 2.2 |  | 0.55 |  | 52 | 73 |  | IL-1β |  | 0.2 |  | 0.53 |  | 51 | 71 |
| CCL4 |  | 23.2 |  | 0.68 |  | 45 | 96 |  | IL-9 |  | 9.3 |  | 0.54 |  | 63 | 60 |  | PDGF |  | 8.1 |  | 0.53 |  | 21 | 100 |
| G-CSF |  | 92.9 |  | 0.66 |  | 49 | 92 |  | CCL3 |  | 2.5 |  | 0.54 |  | 61 | 64 |  | IL-7 |  | 1.0 |  | 0.52 |  | 67 | 57 |
| IL-12 |  | 0.3 |  | 0.64 |  | 99 | 44 |  | IL-7 |  | 1.3 |  | 0.54 |  | 59 | 64 |  | CCL5 |  | 835.9 |  | 0.52 |  | 44 | 71 |
| IL-9 |  | 7.2 |  | 0.64 |  | 56 | 74 |  | IL-17 |  | 3.0 |  | 0.52 |  | 35 | 82 |  | CCL4 |  | 18.2 |  | 0.52 |  | 51 | 71 |
| VEGF |  | 19.8 |  | 0.61 |  | 68 | 57 |  | PDGF |  | 51.5 |  | 0.52 |  | 27 | 91 |  | IL-2 |  | 4.6 |  | 0.51 |  | 88 | 29 |
| IL-2 |  | 3.4 |  | 0.54 |  | 26 | 93 |  | IL-1Ra |  | 81.2 |  | 0.52 |  | 54 | 64 |  | IL-17 |  | 1.6 |  | 0.51 |  | 49 | 71 |
| IL-1Ra |  | 166.8 |  | 0.52 |  | 32 | 87 |  | CCL5 |  | 863.3 |  | 0.52 |  | 58 | 64 |  | IL-13 |  | 2.1 |  | 0.51 |  | 96 | 29 |
| FGF-basic |  | 8.3 |  | 0.52 |  | 18 | 99 |  | VEGF |  | 21.3 |  | 0.51 |  | 30 | 82 |  | G-CSF |  | 674.9 |  | 0.51 |  | 98 | 33 |
| TNF-α |  | 10.7 |  | 0.51 |  | 27 | 97 |  | IL-13 |  | 1.1 |  | 0.51 |  | 88 | 27 |  | CCL3 |  | 268.1 |  | 0.50 |  | 96 | 29 |
| CCL2 |  | 45.6 |  | 0.50 |  | 81 | 4 |  | CXCL8 |  | 1.9 |  | 0.50 |  | 25 | 100 |  | IL-5 |  | 2.2 |  | 0.50 |  | 52 | 71 |

HC = Healthy Controls; nMV = Non-mechanical Ventilation; MV = Mechanical Ventilation; AUC = Area Under the Receiver Operating Characteristic Curve (ROC); Se = Sensitivity; Sp = Specificity.
