# Supplementary material for: Unbalanced networks and disturbed kinetics of serum soluble mediators associated with distinct disease outcomes in severe COVID-19 patients
Source: Front Immunol. 2022 Nov 14;13:1004023. doi: 10.3389/fimmu.2022.1004023 (PMC9701840; doi:10.3389/fimmu.2022.1004023)
Supplement: Supplementary file 2 [file Table_2.docx]

*Supplementary Table 2.* Performance indices of baseline fold changes in serum soluble mediators
(D1-6/D0) to classify patients with SEVERE COVID-19 according to disease outcome

| nVM vs MV | | | | | | | |  | Discharge vs Death | | | | | | | |
| --- | --- | --- | --- | --- | --- | --- | --- | --- | --- | --- | --- | --- | --- | --- | --- | --- |
| Parameters |  | Cut-off |  | AUC |  | Se | Sp |  | Parameters |  | Cut-off |  | AUC |  | Se | Sp |
| **IL-1Ra** |  | **1.3** |  | **0.71** |  | **75** | **75** |  | **IL-7** |  | **0.8** |  | **0.75** |  | **61** | **86** |
| CCL2 |  | 0.5 |  | 0.66 |  | 42 | 100 |  | **IL-13** |  | **1.0** |  | **0.73** |  | **54** | **100** |
| CCL11 |  | 0.9 |  | 0.64 |  | 49 | 86 |  | **PDGF** |  | **1.5** |  | **0.71** |  | **45** | **100** |
| CCL4 |  | 0.6 |  | 0.63 |  | 42 | 100 |  | IL-12 |  | 1.0 |  | 0.64 |  | 52 | 86 |
| IL-12 |  | 1.3 |  | 0.61 |  | 34 | 89 |  | IL-17 |  | 1.6 |  | 0.64 |  | 39 | 100 |
| IFN-γ |  | 0.9 |  | 0.59 |  | 42 | 89 |  | G-CSF |  | 1.6 |  | 0.64 |  | 53 | 100 |
| IL-7 |  | 1.5 |  | 0.59 |  | 37 | 89 |  | IL-2 |  | 1.1 |  | 0.63 |  | 52 | 100 |
| IL-13 |  | 0.3 |  | 0.58 |  | 83 | 44 |  | CCL4 |  | 2.1 |  | 0.62 |  | 36 | 100 |
| TNF-α |  | 0.6 |  | 0.57 |  | 73 | 56 |  | CXCL8 |  | 2.8 |  | 0.61 |  | 36 | 100 |
| IL-5 |  | 1.4 |  | 0.56 |  | 37 | 89 |  | CCL2 |  | 1.8 |  | 0.61 |  | 77 | 67 |
| PDGF |  | 0.7 |  | 0.55 |  | 30 | 100 |  | IL-5 |  | 1.1 |  | 0.61 |  | 51 | 86 |
| CXCL10 |  | 0.2 |  | 0.55 |  | 76 | 44 |  | CCL3 |  | 3.3 |  | 0.6 |  | 34 | 100 |
| IL-2 |  | 1.1 |  | 0.54 |  | 51 | 67 |  | TNF-α |  | 1.7 |  | 0.6 |  | 35 | 100 |
| GM-CSF |  | 1.6 |  | 0.53 |  | 31 | 89 |  | FGF-basic |  | 1.3 |  | 0.59 |  | 35 | 100 |
| IL-10 |  | 0.8 |  | 0.53 |  | 76 | 44 |  | IL-1β |  | 1.3 |  | 0.58 |  | 41 | 100 |
| FGF-basic |  | 1.2 |  | 0.52 |  | 40 | 78 |  | IL-15 |  | 1.4 |  | 0.57 |  | 34 | 100 |
| CCL3 |  | 1.2 |  | 0.52 |  | 49 | 67 |  | IL-4 |  | 1.6 |  | 0.57 |  | 29 | 100 |
| IL-15 |  | 1.2 |  | 0.52 |  | 36 | 86 |  | IL-9 |  | 0.9 |  | 0.57 |  | 69 | 67 |
| G-CSF |  | 1.7 |  | 0.52 |  | 49 | 75 |  | CCL11 |  | 0.4 |  | 0.56 |  | 75 | 50 |
| IL-6 |  | 1.6 |  | 0.51 |  | 37 | 78 |  | GM-CSF |  | 1.6 |  | 0.56 |  | 33 | 100 |
| VEGF |  | 1.1 |  | 0.51 |  | 48 | 78 |  | CCL5 |  | 1.2 |  | 0.53 |  | 66 | 71 |
| IL-9 |  | 1.4 |  | 0.51 |  | 68 | 57 |  | IL-6 |  | 0.4 |  | 0.53 |  | 64 | 57 |
| CCL5 |  | 1.0 |  | 0.51 |  | 59 | 67 |  | VEGF |  | 1.0 |  | 0.53 |  | 41 | 86 |
| IL-17 |  | 1.7 |  | 0.51 |  | 33 | 89 |  | CXCL10 |  | 3.6 |  | 0.52 |  | 93 | 29 |
| IL-1β |  | 1.5 |  | 0.51 |  | 37 | 89 |  | IL-1Ra |  | 1.2 |  | 0.51 |  | 67 | 57 |
| IL-4 |  | 1.6 |  | 0.50 |  | 28 | 89 |  | IL-10 |  | 1.3 |  | 0.51 |  | 80 | 43 |
| CXCL8 |  | 3.0 |  | 0.50 |  | 34 | 89 |  | IFN-γ |  | 0.9 |  | 0.50 |  | 45 | 71 |

HC = Healthy Controls; nMV = Non-mechanical Ventilation; MV = Mechanical Ventilation; AUC = Area Under the Receiver Operating Characteristic Curve (ROC); Se = Sensitivity; Sp = Specificity.
